# Structural basis of negative regulation of CRISPR-Cas7-11 by TPR-CHAT

**DOI:** 10.1038/s41392-024-01821-4

**Published:** 2024-05-13

**Authors:** Tian Hong, Qinghua Luo, Haiyun Ma, Xin Wang, Xinqiong Li, Chongrong Shen, Jie Pang, Yan Wang, Yuejia Chen, Changbin Zhang, Zhaoming Su, Haohao Dong, Xiaodi Tang

**Affiliations:** 1 Department of Laboratory Medicine, State Key Laboratory of Biotherapy, National Clinical Research Center for Geriatrics, West China Hospital, Sichuan University, Chengdu, China; 2 Frontiers Medical Center, Tianfu Jincheng Laboratory, West China Hospital, Sichuan University, Chengdu, China

**Keywords:** Structural biology, Gene therapy

## Abstract

CRISPR‒Cas7-11 is a Type III-E CRISPR-associated nuclease that functions as a potent RNA editing tool. Tetratrico-peptide repeat fused with Cas/HEF1-associated signal transducer (TPR-CHAT) acts as a regulatory protein that interacts with CRISPR RNA (crRNA)-bound Cas7-11 to form a CRISPR-guided caspase complex (Craspase). However, the precise modulation of Cas7-11’s nuclease activity by TPR-CHAT to enhance its utility requires further study. Here, we report cryo-electron microscopy (cryo-EM) structures of *Desulfonema ishimotonii* (*Di*) Cas7-11-crRNA, complexed with or without the full length or the N-terminus of TPR-CHAT. These structures unveil the molecular features of the Craspase complex. Structural analysis, combined with in vitro nuclease assay and electrophoretic mobility shift assay, reveals that *Di*TPR-CHAT negatively regulates the activity of *Di*Cas7-11 by preventing target RNA from binding through the N-terminal 65 amino acids of *Di*TPR-CHAT (*Di*TPR-CHAT_NTD_). Our work demonstrates that *Di*TPR-CHAT_NTD_ can function as a small unit of *Di*Cas7-11 regulator, potentially enabling safe applications to prevent overcutting and off-target effects of the CRISPR‒Cas7-11 system.

## Introduction

The clustered regularly interspaced short palindromic repeats (CRISPR)-Cas system endows bacteria and archaea with adaptive immunity by encoding genes that combat invading exogenous nucleic acids, such as plasmids, phages, and mobile genetic elements.^1–4^ The CRISPR-Cas system has revolutionized the field of genetic engineering and has become a widely used tool in biological research.^5^ Its gene editing capabilities have made it the preferred method for editing genes in a wide range of organisms, including plants, animals, and even human cells.^6,7^

A CRISPR-Cas system consists of two main components: a Cas protein, which is the catalytic core, and CRISPR loci, which have “genetic memory” functions.^8^ Based on the composition of the Cas protein and properties of the effector complexes, CRISPR-Cas systems are classified into Class 1 and Class 2 systems.^9^ The Class 1 system, including Types I, III, and IV, is characterized by an effector complex composed of four to seven Cas protein subunits and mediates foreign nucleic acid interference through the action of multiple Cas protein complexes.^9,10^ In contrast, a Class 2 system, including Types II, V, and VI, carries a single multidomain effector protein.^10^ CRISPR-Cas systems target double-stranded or single-stranded DNA, as well as single-stranded RNA.^10–12^

**Fig. 1:**
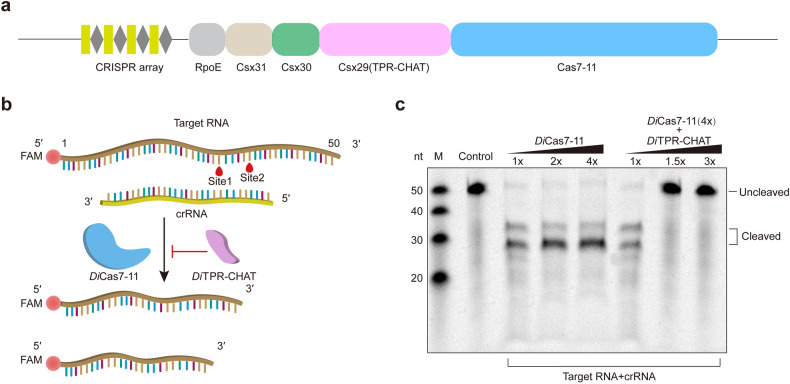
*Di*TPR-CHAT inhibits the cleavage activity of *Di*Cas7-11. **a** A domain structure illustration of the *Di*Type III-E CRISPR-Cas system. **b** Diagram illustrating target RNA cleavage by *Di*Cas7-11-crRNA or inhibition by *Di*TPR-CHAT. **c** In vitro analysis of target RNA cleavage by *Di*Cas7-11 at varying molar concentrations of (1×, 2× or 4×) in the presence of crRNA with or without *Di*TPR-CHAT (1×, 1.5× or 3×) (*n* = 3). The *Di*Cas7-11-crRNA was preincubated with *Di*TPR-CHAT before adding the target RNA. The control consists of the labeled target RNA only

Recently, Cas7-11 (also known as gRAMP) was identified as a novel CRISPR-Cas subtype: Type III-E.^9,13–15^ Cas7-11 is a single effector fusion protein, comprising four Cas7 proteins and one Cas11 protein, but it lacks the signature Cas10 protein common to Type III systems.^13^ Studies have shown that Cas7-11 processes precursor CRISPR RNA (pre-crRNA) and cleaves target RNA at two distinct sites separated by six nucleotides.^13,14^ In contrast to Cas13 family proteins, Type VI effectors that also cleave RNA, Cas7-11 exhibits minimal off-target cleavage activity, high specificity, and low levels of cytotoxicity.^13,16,17^ Importantly, the Type III-E system encodes additional components, including a caspase-like protease named TPR-CHAT (or Csx29), along with three accessory proteins: Csx30, Csx31, and RpoE (Fig. 1a).^13,14^ Functional analyses have demonstrated that Cas7-11 directly interacts with TPR-CHAT to form a CRISPR-guided caspase complex (Craspase), which is considered an active protease involved in immune functions.^14,18–24^ While the Cas7-11 system holds potential as an RNA editing tool, further studies are needed to precisely modulate its cleavage activity for broader applications, especially in identifying endogenous regulators of this system.

We analyzed the available regulatory elements and found that TPR-CHAT shows the potential to be used as an accessory protein for regulating Cas7-11-mediated RNA editing. In this study, we present high-resolution cryo-EM structures of crRNA bound *Di*Cas7-11 (*Di*Cas7-11-crRNA complex) alone or in complex with full length or the N-terminal domain of TPR-CHAT (*Di*Cas7-11-crRNA-TPR-CHAT or *Di*Cas7-11-crRNA-TPR-CHAT_NTD_) and the crRNA bound catalytically inactivated mutant *Di*Cas7-11 (*Di*Cas7-11_Inactive_-crRNA complex). These elucidated structures, combined with biochemical analysis, demonstrated that the NTD fragment of TPR-CHAT functions as a small regulatory unit sufficient to inhibit Cas7-11 activity. Specifically, TPR-CHAT_NTD_ stabilizes the conformation of Cas7-11 and prevents the binding of target RNA. Our results provide implications for understanding the precise regulation of the Cas7-11 system and advancing the application of this system.

## Results

### *Di*TPR-CHAT inhibits the cleavage activity of *Di*Cas7-11

To uncover the mechanism by which *Di*TPR-CHAT inhibits *Di*Cas7-11 cleavage, we first determined the interaction between *Di*TPR-CHAT and *Di*Cas7-11 through pulldown assays. We conducted pulldown assays using N-terminal His-tagged *Di*Cas7-11 and C-terminal Strep-tagged *Di*TPR-CHAT (Supplementary Fig. 1a, b, d, e). Strep-tagged *Di*TPR-CHAT pulled down purified His-tagged *Di*Cas7-11, regardless of whether the proteins were co-expressed or expressed separately, indicating the formation of a stable complex between *Di*Cas7-11 and *Di*TPR-CHAT (Supplementary Fig. 1c, f). Subsequently, we validated the inhibitory effect of *Di*TPR-CHAT on *Di*Cas7-11 using in vitro nuclease assays by incubating the purified *Di*Cas7-11-crRNA with 5’-FAM-labeled target RNA in the presence or absence of *Di*TPR-CHAT (Fig. 1b and Supplementary Fig. 1g). We found that *Di*Cas7-11-crRNA specifically cleaved the target RNA into two fragments, and the presence of *Di*TPR-CHAT inhibited this cleavage (Fig. 1c). The inhibitory effect of *Di*TPR-CHAT is more prominent when preincubating *Di*Cas7-11-crRNA with TPR-CHAT before adding the target RNA (Supplementary Fig. 1g), suggesting that *Di*TPR-CHAT function as a negative regulator by pre-occupying the active site of *Di*Cas7-11.

### Overall structures of *Di*Cas7-11-crRNA, *Di*Cas7-11-crRNA-TPR-CHAT, *Di*Cas7-11-crRNA-TPR-CHAT_NTD_ and *Di*Cas7-11_Inactive_-crRNA complexes

**Fig. 2:**
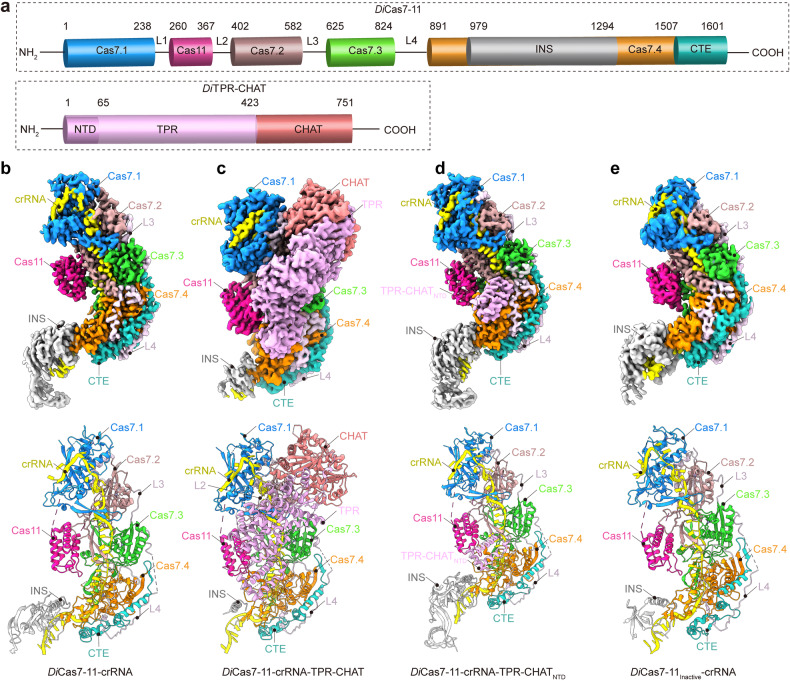
Cryo-EM structures of *Di*Cas7-11-crRNA, *Di*Cas7-11-crRNA-TPR-CHAT, *Di*Cas7-11-crRNA-TPR-CHAT_N**TD,**_ and *Di*Cas7-11_Inactive_-crRNA complexes. **a** Domain structures of *Di*Cas7-11 and *Di*TPR-CHAT. Cryo-EM density map (top) and atomic model (bottom) of the *Di*Cas7-11-crRNA complex (**b**), *Di*Cas7-11-crRNA-TPR-CHAT complex (**c**), *Di*Cas7-11-crRNA-TPR-CHAT _NTD_ complex (**d**), and *Di*Cas7-11_Inactive_-crRNA complex (**e**). The Cas7.1, Cas7.2, Cas7.3, Cas7.4, Cas11, CTE, INS, TPR (TPR-CHAT_NTD_), and CHAT subdomains are depicted in royal blue, magenta, lime green, dark orange, deep pink, light sea green, silver, blue-violet, and light coral, respectively. crRNA is shown in yellow. L2, L3, and L4 are shown in thistle

To investigate the structural basis of the inhibition of *Di*Cas7-11 by *Di*TPR-CHAT, we co-expressed *Di*Cas7-11 and pre-crRNA in the presence or absence of *Di*TPR-CHAT, and purified the *Di*Cas7-11-crRNA and *Di*Cas7-11-crRNA-TPR-CHAT complexes using affinity chromatography and size-exclusion chromatography (Supplementary Fig. 2a, b, d, e, g, h). Cryo-EM was employed to obtain structures of these complexes, with resolutions of 2.93 Å for *Di*Cas7-11-crRNA, 2.90 Å for *Di*Cas7-11-crRNA-TPR-CHAT, 2.86 Å for *Di*Cas7-11-crRNA-TPR-CHAT_NTD_, and 3.53 Å for *Di*Cas7-11_Inactive_-crRNA (Fig. 2a–e and Supplementary Figs. 3–5).

The high-resolution structures unveil atomic details of crRNA-bound *Di*Cas7-11 complexed with full length *Di*TPR-CHAT or *Di*TPR-CHAT_NTD_ (Fig. 2b–d). *Di*Cas7-11 consists of four Cas7 domains (Cas7.1–Cas7.4), a Cas11 domain, and a C-terminal extension (CTE) domain (Fig. 2a–e). Cas7.4 is unique by carrying a large insertion (INS) domain that is absent in the other three Cas7 domains (Fig. 2a–d and Supplementary Fig. 6b). The Cas7 domains, arranged in a row, form a crescent-shaped filament, with the INS and CTE domain completing the structure. The Cas11 domain is positioned at the crescent’s midpoint, and interacts with Cas7.2 and Cas7.3 (Fig. 2b–d and Supplementary Fig. 6c). Cas11 is linked between Cas7.1 and Cas7.2, separated by interspacing linker 1 and 2 (L1 and L2). Cas7.2, Cas7.3 and Cas7.4 are interspaced by L3 and L4. While L1 is disordered in the structures, the remaining linkers are well resolved (Fig. 2a–d and Supplementary Fig. 6a). The crRNA is resolved in all three structures, traversing the Cas7 filament from NTD to the C-terminal domain (CTD) (Fig. 2b–d). The regulatory complex *Di*TPR-CHAT binds to the side of the *Di*Cas7-11 complex, with the CHAT domain interacting with Cas7.1 and Cas7.2, and the NTD of the TPR domain associated at the interface of Cas7.3, Cas7.4 and Cas11 (Fig. 2c and Supplementary Fig. 6d). The truncated TPR-CHAT containing only the NTD can independently interact with the complex at the same position. For obtaining target RNA in the complex, we also obtained the structure using inactivated *Di*Cas7-11-crRNA complex (*Di*Cas7-11_Inactive_) by substituting the conserved catalytic residues, Cas7.2^D429^ and Cas7.3^D654^, with alanine to prevent cleavage of bound target RNA (Fig. 2e and Supplementary Figs. 2c, f, i, 5). However, the resulting complex did not reveal target RNA and exhibited no marked conformational changes to other structures (Fig. 2e and Supplementary Fig. 5).

### Structure comparison of *Di*Cas7-11-crRNA with *Di*Cas7-11-crRNA-TPR-CHAT and *Di*Cas7-11-crRNA-TPR-CHAT_NTD_ complexes

**Fig. 3:**
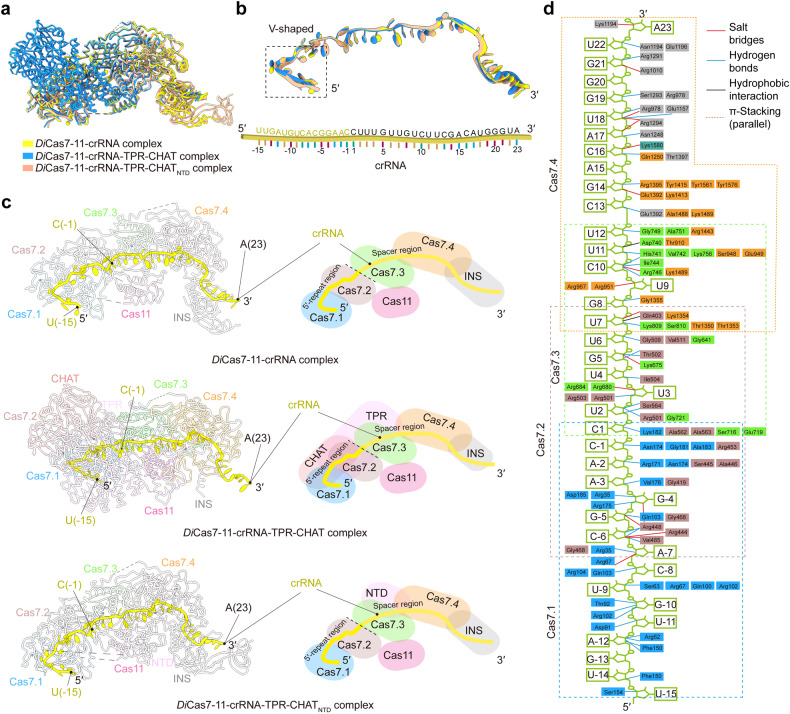
Structural analysis of the *Di*Cas7-11-crRNA, *Di*Cas7-11-crRNA-TPR-CHAT, and *Di*Cas7-11-crRNA-TPR-CHAT_**NTD**_ complexes. **a** Structural superimposition of *Di*Cas7-11-crRNA (yellow), *Di*Cas7-11-crRNA-TPR-CHAT (blue), and *Di*Cas7-11-crRNA-TPR-CHAT_NTD_ (salmon) complexes. **b** Top: The superimposition of the crRNA from the *Di*Cas7-11-crRNA (yellow), *Di*Cas7-11-crRNA-TPR-CHAT (blue), and *Di*Cas7-11-crRNA-TPR-CHAT_NTD_ (salmon) complexes. Bottom: Schematic representation of crRNA in all three structures. **c** Cryo-EM structures and schematic of the *Di*Cas7-11-crRNA, *Di*Cas7-11-crRNA-TPR-CHAT, and *Di*Cas7-11-crRNA-TPR-CHAT_NTD_ complexes, indicating the position of the crRNA (yellow). **d** Interactions between *Di*Cas7-11 and crRNA

The structures of the *Di*Cas7-11-crRNA, *Di*Cas7-11-crRNA-TPR-CHAT, and *Di*Cas7-11-crRNA-TPR-CHAT_NTD_ complexes were superimposed, but no prominent conformational changes were detected (Fig. 3a). The crRNA showed a consistent binding pattern with all three structures (Fig. 3b). Cas7.1 and Cas7.2 anchor the 5′-repeat region (U (−15)–C (−1)) while Cas7.3 and Cas7.4 mainly recognize the spacer region (C (1)–A (23)) of the crRNA (Fig. 3c). Notably, the density of nucleotide U (−16) was absent in all three structures, suggesting that the pre-crRNA had been processed by Cas7-11 into a mature crRNA (U (−15)–A (23)) (Fig. 3b, c). The 5′-repeat region (U (−15)–C (−8)) of the crRNA adopts a ‘V-shaped’ structure and is surrounded by the positively charged residues in the groove of Cas7.1 (Fig. 3b, c and Supplementary Fig. 6e). The interaction between the 5′-repeat region of the crRNA and Cas 7.1 is established mainly by hydrogen bonds (Supplementary Fig. 6f). Specifically, U (−14), A (−12), U (−11), G (−10), and U (−9) form hydrogen bonds with F150, R62/F150, D91, T92/R102, and S63/R67/Q100/R102 of Cas7.1, respectively (Fig. 3d and Supplementary Fig. 6f). These interactions likely contribute to the recognition and processing of the pre-crRNA into mature crRNA. Numerous studies have conducted functional characterizations of the residues that interact with the crRNA in this region, highlighting the essential nature of some amino acid residues for pre-RNA processing.^21,25^ Importantly, in the TPR-CHAT bound structure, the crRNA-binding region retained conformation and exhibited no contact with TPR-CHAT (Fig. 3c, d), indicating that pre-crRNA processing is not mediated by *Di*TPR-CHAT.

*Di*TPR-CHAT consists of two main components: the NTD (amino acid residues 1–65) and the CTD (amino acid residues 75–751) (Fig. 2a). *Di*TPR-CHAT forms multiple interactions with *Di*Cas7-11 through its NTD and CTD (Supplementary Fig. 7a). The NTD interacts with Cas7.4 primarily through hydrogen bonds, salt bridges, and nonbonded contacts (Supplementary Fig. 7a, b). The CTD carries a TPR domain composed of seven TPR units (TPR1–TPR7) and a CHAT protease domain (Supplementary Fig. 7c). The TPR domain of *Di*TPR-CHAT spans from Cas7.1 to Cas7.4, while the CHAT protease domain is close to Cas7.1 and Cas7.2 (Fig. 3c and Supplementary Fig. 6d). The interfaces between the CTD of *Di*TPR-CHAT and *Di*Cas7-11 were mainly formed through nonbonded contacts (Supplementary Fig. 7a). Comparing the structures of *Di*Cas7-11-crRNA-TPR-CHAT and *Di*Cas7-11-crRNA-TPR-CHAT_NTD_, the NTD of *Di*TPR-CHAT was more stabilized in the complex than the CTD, suggesting that TPR-CHAT_NTD_ may play a role in regulating Cas7-11 activity (Fig. 3c).

Our *Di*Cas7-11-crRNA and *Di*Cas7-11-crRNA-TPR-CHAT structures show a high resemblance with a root-mean-square deviation (RMSD) of 0.83 Å between 1118 pruned atom pairs (Supplementary Fig. 8a–c), implicating that the binding of TPR-CHAT did not perturb the conformation of Cas7-11. This characterization is consistent with those recently reported structures from *Desulfonema ishimotonii* (Supplementary Fig. 8d–f)^21^ and *Candidatus ‘Scalindua brodae’* (*Sb*) (Supplementary Fig. 8g–i),^26^ suggesting that the regulation of Cas7-11 by TPR-CHAT may be mediated by minor conformational changes.

### *Di*TPR-CHAT maintains *Di*Cas7-11 in a compact conformation via NTD

**Fig. 4:**
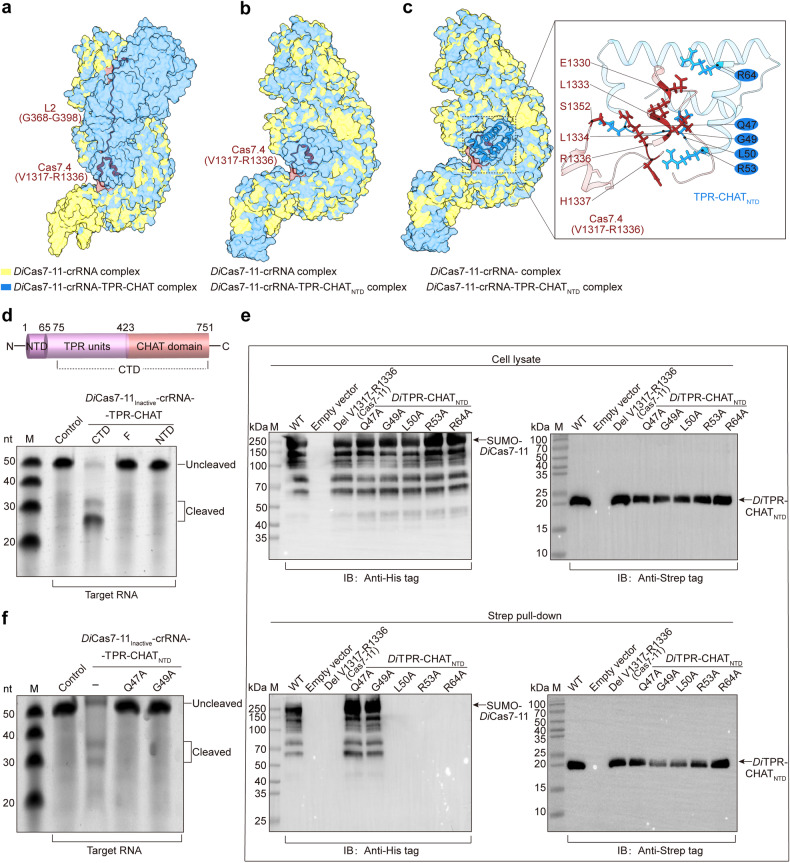
Interaction analysis between *Di*Cas7-11 and *Di*TPR-CHAT or *Di*TPR-CHAT_**NTD**_. **a** Superimposition of the *Di*Cas7-11-crRNA (yellow) and *Di*Cas7-11-crRNA-TPR-CHAT (blue) complexes. L2 (G368-G398) and Cas7.4 (V1371-R1336) in the *Di*Cas7-11-crRNA-TPR-CHAT complex are highlighted in brown. **b** Superimposition of the *Di*Cas7-11-crRNA (yellow) and *Di*Cas7-11-crRNA-TPR-CHAT_NTD_ (blue) complexes. Cas7.4 (V1371-R1336) in the DiCas7-11-crRNA-TPR-CHAT_NTD_ complex is highlighted in brown. **c** The hydrogen bonding interaction between Cas7.4 (V1371-R1336) and NTD in the *Di*Cas7-11-crRNA-TPR-CHAT_NTD_ complex. **d** Top: Domain structures of *Di*TPR-CHAT. Bottom: In vitro- target RNA cleavage analysis catalyzed by the purified complex of *Di*Cas7-11-crRNA-*Di*TPR-CHAT (full length, F), -*Di*TPR-CHAT_CTD_ (CTD), or *Di*TPR-CHAT_NTD_ (NTD) (*n* = 3). The control sample consisted of only labeled target RNA. **e** Pull-down assay between *Di*Cas7-11-crRNA and *Di*TPR-CHAT_NTD_ with different mutations (Cas7.4 carrying segment deletion at V1371-R1336 or single mutation Q47A, G49A, L50A, R53A or R64A in NTD) (*n* = 3). Top: Western-blot analyses of the cell lysates. Bottom: Western-blot analyses of the pulled samples. **f** In vitro target RNA cleavage analysis catalyzed by the purified complex of *Di*Cas7-11-crRNA- *Di*TPR-CHAT_NTD_ (Q47A or G49A). The control sample consists of only the labeled target RNA

To further clarify the negative regulatory mechanism of *Di*Cas7-11 by *Di*TPR-CHAT, we analyzed the above three structures in detail. When the *Di*Cas7-11-crRNA complex bound to *Di*TPR-CHAT or *Di*TPR-CHAT_NTD_, the conformation of the *Di*Cas7-11-crRNA complex was unchanged (Fig. 4a, b). However, the densities for L2 (G368-G398) (in the *Di*Cas7-11-crRNA-TPR-CHAT structure) and the segment between V1317-R1336 in Cas7.4 (both in the *Di*Cas7-11-crRNA-TPR-CHAT and *Di*Cas7-11-crRNA-TPR-CHAT_NTD_ structures) became unambiguous (Fig. 4a, b), suggesting that the binding of *Di*TPR-CHAT_NTD_ stabilizes the conformation of *Di*Cas7-11 in these regions. The enhanced stability of Cas7.4 (V1317-R1336) is attributed to the formation of numerous interactions with *Di*TPR-CHAT_NTD_ (Fig. 4c and Supplementary Fig. 7b). These interactions include a pair of salt bridges formed between Cas7.4 E1330 and TPR-CHAT_NTD_ R64, four pairs of hydrogen bonds formed between Cas7.4 L1333 and TPR-CHAT_NTD_ L50, Cas7.4 L1334, and TPR-CHAT_NTD_ R53, and Cas7.4 E1330 and TPR-CHAT_NTD_ R64 (two pairs), and dozens of pairs of nonbonded contacts (Fig. 4c and Supplementary Fig. 7b). Interestingly, the isolated NTD fragment of *Di*TPR-CHAT was still capable to inhibit the nuclease activity of *Di*Cas7-11 while the CTD fragment was not, suggesting the regulatory activity of *Di*TPR-CHAT was retained in the NTD (Fig. 4d and Supplementary Fig. 9a, b, f).

We further performed a pulldown assay to analyze the interactions between *Di*Cas7.4 and *Di*TPR-CHAT_NTD_. Deletion of V1317-R1336 in Cas7.4 disrupted complex formation between *Di*Cas7-11 and *Di*TPR-CHAT_NTD_. Similarly, mutations of the interacting residues L50A, R53A, and R64A of *Di*TPR-CHAT_NTD_ at the interface also disrupted the complex formation with *Di*Cas7-11 (Fig. 4e and Supplementary Fig. S7b). In contrast, the mutations of the NTD residues Q47A and G49A that formed hydrogen bonds with S1352 and H1337, respectively, outside of the segment of V1317-R1336 in Cas7.4 neither affected the formation of the complex nor the inhibitory activity of *Di*TPR-CHAT_NTD_ (Fig. 4e, f and Supplementary Fig. S7b). These suggest that *Di*TPR-CHAT_NTD_ regulates the activity of *Di*Cas7-11 primarily through the segment of V1317-R1336 in Cas7.4. A recent study also demonstrated the regulatory function of truncated NTD of *Dm*TPR-CHAT on *Dm*Cas7-11 through interactions with the insertion finger in Cas7.4 (Supplementary Fig. 11a–c).^18^ This similar interactive mechanism indicates the importance of TPR-CHAT_NTD_ for Cas7-11 regulation, although the sequences and structures of *Di*TPR-CHAT_NTD_ and *Dm*TPR-CHAT_NTD_ are poorly conserved (Supplementary Fig. 11d–g). Surprisingly, the same mutations carried out on the full length *Di*TPR-CHAT did not affect the formation of complexes with *Di*Cas7-11 or the inhibitory effect of *Di*TPR-CHAT (Supplementary Fig. 10a–d). This suggests that the full length *Di*TPR-CHAT also interacts with Cas7-11 through interfaces other than that between Cas7.4 and *Di*TPR-CHAT_NTD_. Indeed, the structure of *Di*Cas7-11-crRNA-TPR-CHAT also exhibits interactions between L2 and CHAT domains in additional to those in the NTD (Supplementary Fig. S7a), which explains the stabilized conformation of L2 observed in the structure.

### *Di*TPR-CHAT_NTD_ inhibits the binding of target RNA to *Di*Cas7-11-crRNA

**Fig. 5:**
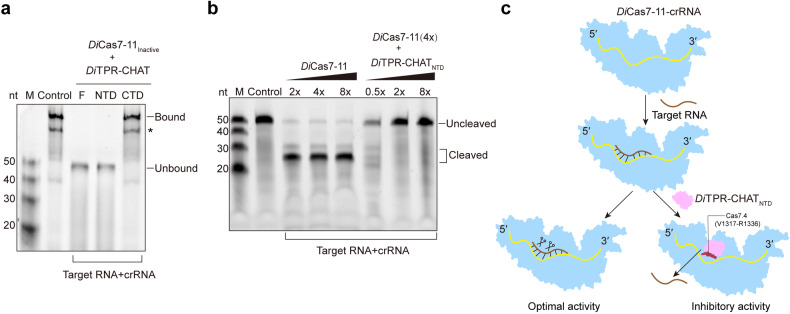
The mechanism by which *Di*TPR-CHAT_N**TD**_ inhibits the activity of *Di*Cas7-11. **a** EMSA of target *Di*Cas7-11_Inactive_ with *Di*TPR-CHAT (Full length, F), *Di*TPR-CHAT_NTD_ (NTD), and *Di*TPR-CHAT_CTD_ (CTD) in the presence of crRNA and labeled target RNA (*n* = 3). The control sample consisted of *Di*Cas7-11_Inactive_, crRNA and target RNA. The bands indicated by an asterisk (*) is a non-specific band that appear constantly in EMSA, which may be from the degradation of target RNA. **b** In vitro target RNA cleavage assay by crRNA-*Di*Cas7-11 (at 2×, 4× or 8× molar concentrations) preincubated with *Di*TPR-CHAT_NTD_ (at 0.5×, 2× or 8× molar concentrations). The control sample consisted of only labeled target RNA. **c** Model of *Di*Cas7-11 showing inhibition by *Di*TPR-CHAT_NTD_. In the absence of *Di*TPR-CHAT_NTD_, *Di*Cas7-11 adopts a relaxed conformation and exhibits optimal cleavage activity. When bound to *Di*TPR-CHAT_NTD_, *Di*Cas7-11 presents a compact conformation (Cas7.4 (V1371-R1336) is visible), blocking target RNA from binding and reducing cleavage activity

It is reported that the RNA cleavage site of *Di*Cas7-11 is situated around Cas7.2 and Cas7.3.^13,14^ Therefore, the modulation of *Di*TPR-CHAT_NTD_ in Cas7.4 would more likely impact the binding but not the cleavage of target RNA. To test this, we expressed and evaluated the affinity between the labeled target RNA and the inactivated *Di*Cas7-11 (mutated catalytic residues at D429/D654) in the presence or absence of the full length, NTD or CTD of TPR-CHAT using electrophoretic mobility shift assays (EMSA) (Supplementary Fig. 9c, d, f). We found that the affinity of the *Di*Cas7-11-crRNA complex to the target RNA was reduced by the presence of full length (F) or NTD of *Di*TPR-CHAT but unaffected by the CTD (Fig. 5a and Supplementary Fig. 1h). These results demonstrated that both *Di*TPR-CHAT and *Di*TPR-CHAT_NTD_ blocked the binding of the target RNA to *Di*Cas7-11, indicating that binding of *Di*TPR-CHAT, mainly via its NTD, reduced target RNA binding and inhibited cleavage activity, probably through modulating the conformation of the *Di*Cas7-11-crRNA complex. *Di*TPR-CHAT_NTD_ was able to inhibit the activity of Cas7-11 in a concentration-dependent manner (Fig. 5b and Supplementary Fig. 9e, g), indicating that the NTD of *Di*TPR-CHAT is sufficient to act as an inhibitory regulator for the activity of *Di*Cas7-11. Based on these findings, we propose a model for the regulation of *Di*Cas7-11 by *Di*TPR-CHAT that *Di*Cas7-11 in a relaxed conformation initially binds to crRNA, which then facilitates the binding of the target RNA to crRNA for cleavage (Fig. 5c). When *Di*TPR-CHAT binds to this Cas7-11–crRNA complex, it stabilizes *Di*Cas7-11-crRNA, primarily through *Di*TPR-CHAT_NTD_, causing the complex to adopt a compact conformation (Fig. 5c). This conformational change inhibited the binding of target RNA, leading to the reduced nuclease activity (Fig. 5c).

## Discussion

The type III-E CRISPR-Cas system is a newly identified bifunctional complex with nuclease activity and protease activity that holds great potential for RNA editing applications.^9,15^ Previous studies on this system have focused more on the mechanism of RNA recognition and cleavage by Cas7-11 and the mechanism of substrate protein cleavage by TPR-CHAT induced by target RNA binding.^19–24,26–28^ However, the poor understanding of the precise modulation of Cas7-11 by TPR-CHAT limits the application of the system.

In this study, we first demonstrated that *Di*Cas7-11 possesses cleavage activity towards target RNA guided by crRNA and that *Di*TPR-CHAT can inhibit the cleavage activity of *Di*Cas7-11. To gain further insights into the mechanism of *Di*TPR-CHAT regulation of *Di*Cas7-11, we determined the high-resolution structures of the *Di*Cas7-11-crRNA and *Di*Cas7-11-crRNA-TPR-CHAT complexes (both full-length and N-terminal domain of *Di*TPR-CHAT) using cryo-EM. Structural analysis revealed that the binding of *Di*TPR-CHAT did not induce significant conformational changes in the overall configuration of *Di*Cas7-11-crRNA complex, but it did stabilize the conformations in the L2 and Cas7.4 regions. Further analysis demonstrated that *Di*TPR-CHAT, primarily via its NTD, maintained the stable conformation of *Di*Cas7-11-crRNA by establishing multiple interactions with Cas7.4. This contributed to the prevention of target RNA binding to the crRNA and resulted in reduced cleavage activity. These findings are consistent with the studies by Huo et al. (Supplementary Fig. 12) and Babatunde Ekundayo et al. (Supplementary Fig. 11a–c), which suggest that the regulatory mechanism may be conserved among different species.^18,21^

The regulatory characterization of *Di*TPR-CHAT_NTD_ by preventing target RNA binding to Cas protein is similar to anti-CRISPR proteins (Acrs).^8,29–32^ It is a class of small protein inhibitors that target the CRISPR-Cas system and play a crucial role in precisely regulating the CRISPR-Cas to reduce off-target effects and cell cytotoxicity.^33–35^ Although the current study on the *Di*Cas7-11 system shows little off-target effects,^13^ it is important to consider the potential occurrence of off-target effects as the system becomes more widely used, which could impact its safety. Therefore, investigating inhibitors that target *Di*Cas7-11 is necessary. *Di*TPR-CHAT_NTD_, a truncated form of *Di*TPR-CHAT with a smaller molecular weight but similar function, represents an ideal protein inhibitor against *Di*Cas7-11. It holds significant potential for various applications in gene-editing technology. However, it is noteworthy that the activity of *Di*TPR-CHAT_NTD_ has only been demonstrated in vitro, and whether it can exert a similar inhibitory effect in vivo, particularly in mammalian cells, requires further study. Thus, the development of TPR-CHAT_NTD_ as a regulator to control Cas7-11 activity biologically requires additional refinement.

In summary, our findings highlight the role of *Di*TPR-CHAT_NTD_ in negatively regulating the nuclease activity of *Di*Cas7-11 by blocking the binding of target RNA. Understanding the negative regulation of *Di*Cas7-11 by *Di*TPR-CHAT is crucial for deciphering the complex dynamics of bacterial immune responses and may have implications for the development of novel strategies to modulate CRISPR-Cas activity. Future research efforts will focus on adapting and optimizing this system for use in RNA editing, with the goal of developing it as a therapeutic tool and gene detection application for clinical use.

## Materials and methods

### Plasmid construction

The codon-optimized sequence of *DiCas7-11* (encoding amino acid residues 1–1601) was amplified by PCR and inserted into a modified pET28a vector that contained an N-terminal eight-histidine (8×His) tag followed by a small ubiquitin-like modifier (SUMO)-fusion sequence and a tobacco etch virus (TEV) protease cleavage site (pET28a-His-SUMO-TEV-*Di*Cas7-11). To construct a crRNA-expressing plasmid, a single spacer pre-crRNA sequence comprising six direct repeats and five spacers was cloned at multiple cloning site 2 (MCS2) of a pACYCDuet-1 vector (pACYCDuet-1-crRNA). The codon-optimized sequences of *DiTPR-CHAT* (encoding amino acid residues 1–751) and *DiTPR-CHAT*_*NTD*_ (encoding amino acid residues 1–65) carrying a 2×Strep tag at the C-terminal domain (CTD) were amplified by PCR and inserted into the pET28a-His-SUMO-TEV-*Di*Cas7-11 vector (pET28a-His-SUMO-TEV-*Di*Cas7-11-TPR-CHAT-2×Strep and pET28a-His-SUMO-TEV-*Di*Cas7-11-TPR-CHAT_NTD_-2×Strep). To generate an inactivated *Di*Cas7-11 variant to prevent catalysis of the bound target RNA, two conserved catalytic residues in Cas7, Asp429 and Asp654, were mutated to an alanine residue (pET28a-His-SUMO-TEV-*Di*Cas7-11_Inactive_) using the method described previously.^36^

### Expression and purification of *Di*Cas7-11-crRNA, *Di*Cas7-11-crRNA-TPR-CHAT and *Di*Cas7-11_Inactive_-crRNA complexes

For the expression and purification of the *Di*Cas7-11-crRNA complex, the constructed pET28a-His-SUMO-TEV-*Di*Cas7-11 and pACYCDuet-1-crRNA plasmids were co-transformed into the *E. coli* BL21 (DE3) strain. The bacterial cells were grown overnight in LB medium supplemented with 50 μg ml-1 kanamycin and 25 μg ml^−1^ chloramphenicol at 37 °C. Protein expression was induced with 0.5 mM isopropyl β-D-thiogalactoside (IPTG) by incubating the cells at 18 °C for 14–16 h, at which time the optical density (OD) of the culture at 600 nm reached 0.8. Cell pellets were harvested by centrifugation, resuspended in buffer A (50 mM HEPES-NaOH, pH 8.0, 500 mM NaCl, and 0.5 mM dithiothreitol (DTT)), and then supplemented with 1 mM phenylmethylsulfonyl fluoride (PMSF). The cells were lysed, and the supernatant was harvested following centrifugation at 18,000 × *g* for 45 min at 4 °C. The supernatant harboring the Cas7-11-crRNA complex was purified using a nickel-nitrilotriacetic acid (Ni-NTA) column (GE Healthcare) with buffer A containing 250 mM imidazole. The eluted protein was further purified using a Superdex 200 Increase 10/300 column (GE Healthcare) pre-equilibrated with buffer C (20 mM HEPES-NaOH, pH 8.0, 300 mM NaCl, and 0.5 mM DTT). The peak fractions were collected for subsequent cryo-EM sample preparation or stored at −80 °C until further use.

For the expression and purification of the *Di*Cas7-11-crRNA-TPR-CHAT complex, the two prepared plasmids (pET28a-His-SUMO-TEV-*Di*Cas7-11-TPR-CHAT-2×Strep and pACYCDuet-1-crRNA) were co-transformed into the *E. coli* BL21 (DE3) strain. A single colony was grown in LB medium supplemented with 50 μg ml^−1^ kanamycin and 25 μg ml^−1^ chloramphenicol at 37 °C. Protein expression was induced by 0.5 mM IPTG at 18 °C for 14–16 h until the OD 600 nm reached 0.8. Cell pellets were then collected by centrifugation and resuspended in buffer A supplemented with 1 mM PMSF. The cells were lysed and the supernatant was collected after centrifugation at 18,000 × *g* for 45 min at 4 °C. The supernatant containing the *Di*Cas7-11-crRNA-TPR-CHAT complex was purified using a Ni-NTA column (GE Healthcare) in buffer A supplemented with 250 mM imidazole. The eluted protein was further purified by chromatography on a HiTrap Strep column system (GE Healthcare) equilibrated with buffer B. The protein was eluted with 10 mM D-desthiobiotin (Sigma) and then purified on a Superdex 200 Increase 10/300 column (GE Healthcare) pre-equilibrated with buffer C. The collected peak fractions were designated for cryo-EM sample preparation or stored at −80 °C until required.

For the expression and purification of the *Di*Cas7-11_Inactive_-crRNA complex, the constructed plasmid (pET28a-His-SUMO-TEV-*Di*Cas7-11_Inactive_) was transformed into the *E. coli* BL21 (DE3) strain and cultivated overnight in LB medium containing 50 μg ml^−1^ kanamycin. A single colony was cultured in LB medium containing 50 μg ml^−1^ kanamycin at 37 °C. Protein expression was induced by adding 0.5 mM IPTG and maintained at 18 °C for 14–16 h until the OD600 nm reached 0.8. Cell pellets were harvested after centrifugation and resuspended in buffer A containing 1 mM PMSF. Cells were lysed and the supernatant harvested by centrifugation at 18,000 × *g* for 45 min at 4 °C. The supernatant was purified using a Ni-NTA column (GE Healthcare) with buffer A containing 250 mM imidazole. The eluted protein was further purified by chromatography on a HiTrap Heparin column (GE Healthcare) equilibrated with buffer B. The protein was eluted with a linear gradient of 0–2 M NaCl. The peak fractions were collected and further purified on a Superdex 200 Increase 10/300 column (GE Healthcare) equilibrated with buffer C. For cryo-EM sample preparation, the purified *Di*Cas7-11_Inactive_ sample was incubated with single spacer pre-crRNA (5’-GGUUGGAAAGCCGGUUUUCUUUGAUGUCACGGAACCUUUGUUGUCUUCGACAUGGGUAAUCCUCAU-3’) at a molar ratio of 1:2 for 1 h at room temperature.

### In vitro nuclease assays

In vitro nuclease assays were carried out using purified *Di*Cas7-11-crRNA- *Di*TPR-CHAT complex (for fixed-dose at 233 nM) or individually purified *Di*Cas7-11-crRNA preincubated with *Di*TPR-CHAT (for increasing doses) in a nuclease assay buffer (40 mM Tris-HCl, pH 7.5, 60 mM NaCl, and 6 mM MgCl_2_) supplemented with 4 U of an RNase inhibitor. 10 nM 5-carboxyfluorescein (5’-FAM)-labeled target RNA was added to the system and incubated at 37 °C for 30 min. Reactions with different assembly orders of the components were also carried out. To terminate the reactions, 1.5 mg ml^−1^ proteinase K, 6 mM EDTA, and 500 mM urea were added and incubated at 50 °C for 30 min. The samples were then heated at 95 °C for 3 min and subjected to electrophoresis on a urea gel at 180 V for 35 min at room temperature. The gels were scanned using an iBright FL1500 Imaging System (Thermo Fisher Scientific).

### Protein pulldown assays

To characterize the interaction between *Di*Cas7-11 and *Di*TPR-CHAT in vivo, the pET28a-His-SUMO-TEV-*Di*Cas7-11-TPR-CHAT-2×Strep plasmid was transformed into the *E. coli* BL21 (DE3) strain and cultured overnight in LB medium supplemented with 50 μg ml^−1^ kanamycin. A single colony was cultured in LB medium supplemented with 50 μg ml^−1^ kanamycin at 37 °C. The protein overexpression and purification protocols were performed as previously described. The complex was identified via SDS‒PAGE and Coomassie Brilliant Blue staining. To identify the interaction between *Di*Cas7-11 and *Di*TPR-CHAT in vitro, the codon-optimized sequence of *Di*TPR-CHAT (residues 1–751), carrying a 2×Strep tag at the CTD, was amplified by PCR and cloned into the pET28a vector (pET28a-*Di*TPR-CHAT-2×Strep). The plasmids pET28a-His-SUMO-TEV-*Di*Cas7-11 and pET28a-*Di*TPR-CHAT-2×Strep were separately transformed into the *E. coli* BL21 (DE3) strain and cultured overnight in LB medium supplemented with 50 μg ml^−1^ kanamycin. A single colony from each transformation was cultured in individual LB medium containing 50 μg ml^−1^ kanamycin at 37 °C. The expression of *Di*Cas7-11 and *Di*TPR-CHAT was induced with IPTG, followed by their purification using a Ni-NTA column (GE Healthcare) and a HiTrap Strep column (GE Healthcare), respectively. Imidazole was removed using a desalting column (Bio-Rad). His-tagged *Di*Cas7-11 and Strep-tagged *Di*TPR-CHAT were co-incubated at 4 °C for 1 h at a 1:1 molar ratio. The mixtures were then re-purified using a Ni-NTA column (GE Healthcare) and HiTrap Strep column (GE Healthcare). The eluted protein was further purified on a Superdex 200 Increase 10/300 column (GE Healthcare), and the peak fraction was collected for SDS‒PAGE and Coomassie Brilliant Blue staining.

To determine whether *Di*TPR-CHA or *Di*TPR-CHAT_NTD_ interacts with *Di*Cas7-11through the region V1317-R1336, the plasmid pET28a-His-SUMO-TEV-*Di*Cas7-11-TPR-CHAT-2×Strep or pET28a-His-SUMO-TEV-*Di*Cas7-11-TPR-CHAT_NTD_-2×Strep was utilized as a template for deleting the segment V1317-R1336 in Cas7-11. The resulting mutagenesis plasmids and pACYCDuet-1-crRNA plasmid were co-transformed into the *E. coli* BL21 (DE3) strain. The pACYCDuet-1 plasmid and pET28a-His-SUMO plasmid were also co-transformed as negative controls. A single colony was cultured in LB medium supplemented with 50 μg ml^−1^ kanamycin and 25 μg ml^−1^ chloramphenicol and incubated at 37 °C. Protein expression was induced by adding 0.5 mM IPTG until the OD600 nm reached 0.8, and the cells were cultured at 18 °C for 14–16 h. Cells were harvested and lysed by sonication. The expression of wild-type or *Di*Cas7-11 (del V1317-R1336) and *Di*TPR-CHAT/*Di*TPR-CHAT_NTD_ were detected by immunoblotting using mouse anti-His monoclonal antibody (1:1000 dilution; SAB2702218, Sigma) and mouse anti-Strep monoclonal antibody (1:1000 dilution; SAB2702215, Sigma), respectively. Protein targets were visualized by chemiluminescence using horseradish peroxidase-conjugated rabbit anti-mouse antibody (1:5000 dilution; A9044, Sigma) and imaged with a photo imager (GE Healthcare). The proteins purified by the HiTrap Strep column were tested for interactions by the method described above.

### Electrophoretic mobility shift assay (EMSA)

Purified *Di*Cas7-11_Inactive_-crRNA-TPR-CHAT, *Di*Cas7-11_Inactive_-crRNA-TPR-CHAT_NTD_, and *Di*Cas7-11-crRNA-TPR-CHAT_CTD_ complexes (233 nM each) were incubated with 30 nM 5’-FAM-labeled target RNA in buffer containing 40 mM Tris-HCl, pH 7.5, 60 mM NaCl, and 6 mM MgCl_2_ for 30 min at 4 °C. Reactions of the individually purified components mixed at different assembly orders were also carried out. The samples were then loaded onto a 15% native PAGE gel and separated at 250 V for 2.5 h. The fluorescence signal was captured using an iBright FL1500 Imaging System (Thermo Fisher Scientific).

### Cryo-EM sample preparation and data acquisition

For cryo-EM grid preparation, 3.0 μL of purified *Di*Cas7-11-crRNA, *Di*Cas7-11_Inactive_-crRNA, and *Di*Cas7-11-crRNA-TPR-CHAT complexes were applied to glow-discharged (45 s) holey carbon grids (Quantifoil Au R2/1, 200 mesh). The grids were blotted for 3.0 s and then frozen by plunging into liquid ethane that had been cooled with liquid nitrogen using a Vitrobot Mark IV system (Thermo Fisher Scientific) at 4 °C and 100% humidity. The grids were loaded onto a Titan Krios cryogenic electron microscope (Thermo Fisher Scientific) operating at 300 kV. The microscope equipped with a 50 μm condenser lens aperture was set to a spot size of 6 and magnification at 165,000× (corresponding to a calibrated sample at 0.85 Å per physical pixel) and a K2 direct electron device equipped with a BioQuantum energy filter operated at 20 eV (Gatan). Movie stacks were collected automatically using EPU2 software (Thermo Fisher Scientific) with the K2 detector operating in counting mode. The recording rate was set to 5 raw frames per second, and the total exposure time was set to 6 s, resulting in 30 frames per stack and a total dose of ~60 e–/Å2. A total of 3232 movie stacks were collected for the *Di*Cas7-11-crRNA-TPR-CHAT complex, 2997 movie stacks were collected for *Di*Cas7-11-crRNA, and 1913 movie stacks were collected for *Di*Cas7-11_Inactive_-crRNA. The defocus range during data collection spanned from −1.0 to −1.8 μm.

### Image processing and 3D image reconstruction

Cryo-EM data processing was conducted using EMAN (v2.31),^37^ Relion (v4.0) and cryoSPARC (v4.1).^38,39^ For the *Di*Cas7-11-crRNA-TPR-CHAT complex, a total of 3232 movie stacks were collected. All 30 frames in each stack were added with motion correction using MotionCorr2^40^ and double binning to attain a pixel size of 0.85 Å per pixel. Dose weighting was also applied. Visual inspection of each micrograph was conducted to remove poor-quality images due to contamination from crystalline ice or other visible contaminants. After contrast transfer function (CTF) correction using CTFFIND4,^41^ 3228 micrographs were processed for neural network particle picking in EMAN2.2. A total of 622,076 particles were isolated in Relion4 using a 320 pixel box. The extracted particles were downscaled twice and subjected to reference-free 2D classification to eliminate false picks and obvious junk classes. After two rounds of 2D classification, approximately 499,545 particles in the best classes were visually examined and selected for further analysis. These particles were then input into cryoSPARC to generate an initial model. All particles were divided into six classes, and two classes showed distinct protein profiles after ab initio reconstruction and heterogeneous refinement in cryoSPARC. One class contained 78,984 particles (15.8% of the total), and the other class contained 147,448 particles (29.5% of the total). These two classes were imported into Relion for autorefinement and Bayesian polishing. Finally, 3D nonuniform refinement and local refinement options in cryoSPARC were employed to determine the global resolution of the *Di*Cas7-11-crRNA-TPR-CHAT map (2.90 Å) and *Di*Cas7-11-crRNA-TPR-CHAT_NTD_ map (2.86 Å). The resolutions for these final maps were estimated using the 0.143 criterion of the Fourier shell correlation (FSC) curve in cryoSPARC. The final maps were subjected to low-pass filtering and visualized in UCSF Chimera.^42^

For the *Di*Cas7-11-crRNA complex, a total of 2997 movies were collected. From these movies, 529,643 particles were extracted using cryoSPARC and categorized into six classes for ab initio reconstruction and heterogeneous refinement. Of these, 216,156 particle projections (representing 40.8% of the total) from the selected optimal class were processed through final homogeneous refinement, nonuniform refinement, and local refinement. The resulting density map exhibited a global resolution of 2.93 Å.

For the *Di*Cas7-11_Inactive_-crRNA complex, a total of 1913 movies were collected. CryoSPARC was used to extract 566,672 particles, which were then classified into five classes for ab initio reconstruction and heterogeneous refinement. Among these, 106,871 particle projections (50.1% of the total) from the optimal class were subjected to further processing, including focused 3D classification in Relion. Ultimately, 21,836 particles were selected for nonuniform refinement and local refinement in cryoSPARC. The resulting density map showed a global resolution of 3.53 Å.

### Model building and structure refinement

The initial models for the *Di*Cas7-11-crRNA-TPR-CHAT, *Di*Cas7-11-crRNA-TPR-CHAT_NTD_, and *Di*Cas7-11-crRNA complexes were constructed based on a previously reported structure (PDB ID 7Y9X).^23^ For the *Di*Cas7-11_Inactive_-crRNA complex, the initial model was derived from another structure (PDB ID 7WAH).^25^ These initial models were used for model reconstruction and refinement in conjunction with the cryo-EM density maps. The docking of these models incorporated with the EM density maps was performed using UCSF Chimera.^42^ Subsequently, manual adjustment and model rebuilding were carried out using COOT (v0.9.6).^43^ These models were further refined using Phenix.real_space_refine (v1.19.2-4158).^44^ The refinement process resulted in average model–map correlation coefficients (CCmask) of 0.82, 0.80, 0.70, and 0.76 for the *Di*Cas7-11-crRNA-TPR-CHAT, *Di*Cas7-11-crRNA-TPR-CHAT_NTD_, *Di*Cas7-11-crRNA, and *Di*Cas7-11_Inactive_-crRNA complexes, respectively. The validity of the model structures was assessed using MolProbity.^45^ The final model structures exhibited reasonable geometry. The statistical analyses related to the refinement steps are provided in Table S1.

### Supplementary information

Supplementary figures

## Data Availability

EM density maps and atomic models have been deposited in the Electron Microscopy Data Bank (EMDB) and PDB, respectively, with accession codes EMDB-37640 and 8WM4 (*Di*Cas7-11-crRNA complex), EMDB-37649 and 8WMC (*Di*Cas7-11-crRNA-TPR-CHAT complex), EMDB-37655 and 8WML (*Di*Cas7-11-crRNA-TPR-CHAT_NTD_ complex), EMDB-37653 and 8WMI (*Di*Cas7-11_Inactive_-crRNA complex).
