# Supplementary material for: Structural basis of negative regulation of CRISPR-Cas7-11 by TPR-CHAT
Source: Signal Transduct Target Ther. 2024 May 13;9:111. doi: 10.1038/s41392-024-01821-4 (PMC11089037; doi:10.1038/s41392-024-01821-4)
Supplement: Supplementary file 1 — Supplementary figures [file 41392_2024_1821_MOESM1_ESM.pdf]

Supplementary Materials for

Structural basis of negative regulation of CRISPR-Cas7-11 by TPR-CHAT

Tian Hong†, Qinghua Luo†, Haiyun Ma†, Xin Wang†, Xinqiong Li†, Chongrong Shen, Jie Pang, Yan Wang, Yuejia Chen, Changbing Zhang, Zhaoming Su*, Haohao Dong*, Xiaodi Tang*

*Correspondence: Zhaoming Su (zsu@scu.edu.cn) or Haohao Dong (haohaodong@scu.edu.cn) or Xiaodi Tang (tangxiaodi@scu.edu.cn)

This PDF file includes:

Figures. S1 to S12

Table S1

The original uncropped Western blots

Figure. S1.

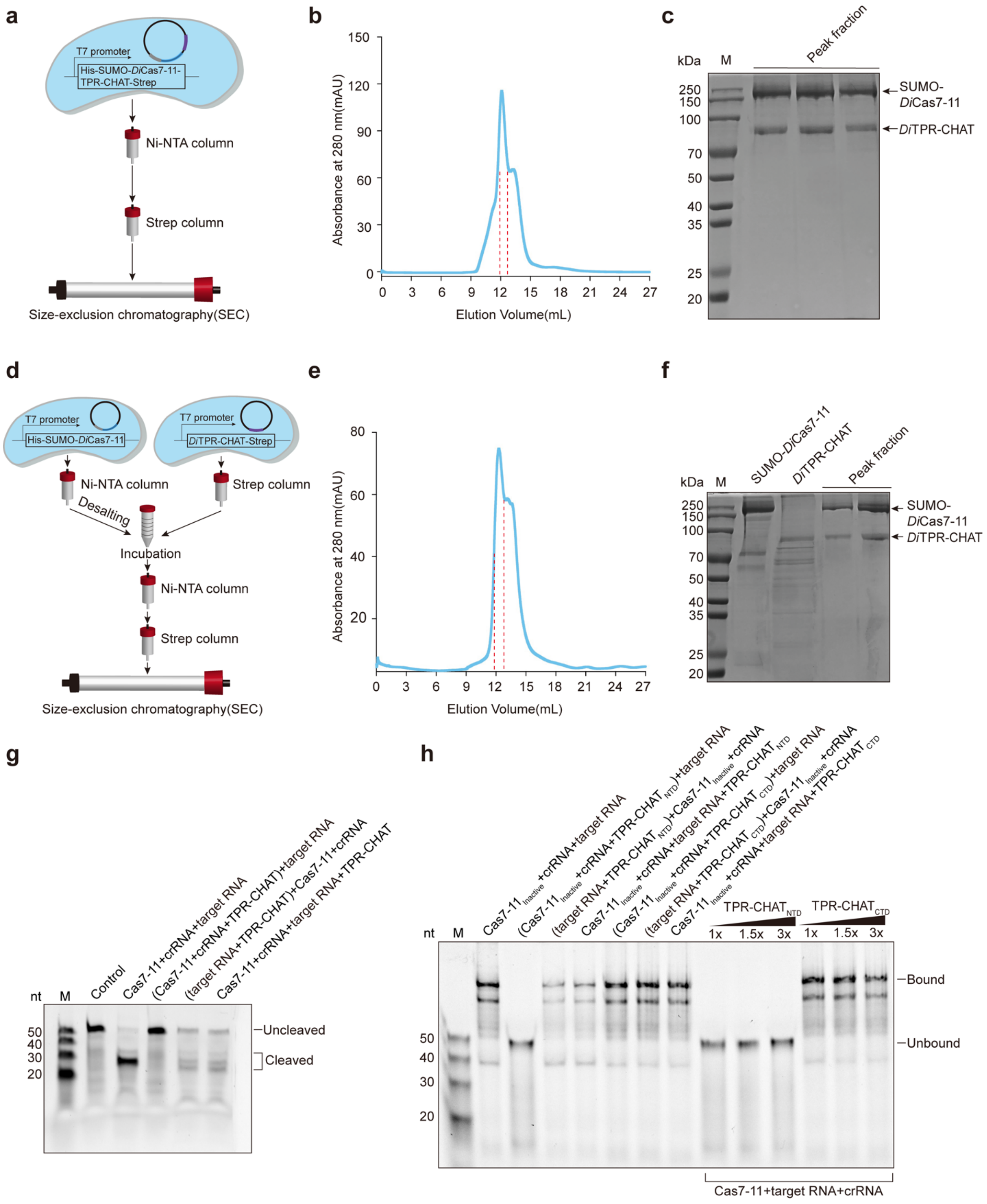
a
T7 promoter
His-SUMO-DiCas7-11-TPR-CHAT-Strep
Ni-NTA column
Strep column
Size-exclusion chromatography(SEC)
b
Absorbance at 280 nm(mAU)
Elution Volume(mL)
c
kDa
M
Peak fraction
SUMO-DiCas7-11
DiTPR-CHAT
d
T7 promoter
His-SUMO-DiCas7-11
DiTPR-CHAT-Strep
Ni-NTA column
Strep column
Desalting
Incubation
Size-exclusion chromatography(SEC)
e
Absorbance at 280 nm(mAU)
Elution Volume(mL)
f
SUMO-DiCas7-11
DiTPR-CHAT
Peak fraction
g
nt
M
Control
Cas7-11+crRNA+target RNA
(Cas7-11+crRNA+TPR-CHAT)+target RNA
(target RNA+TPR-CHAT)+Cas7-11+crRNA
Cas7-11+crRNA+target RNA+TPR-CHAT
Uncleaved
Cleaved
h
Bound
Unbound
TPR-CHAT NTD
TPR-CHAT CTD
1x
1.5x
3x
Cas7-11+target RNA+crRNA

Figure. S1. Pulldown assays of *Di*Cas7-11 and *Di*TPR-CHAT in vivo and in vitro. **a** Workflow of the pulldown assay of *Di*Cas7-11 and *Di*TPR-CHAT in vivo. **b** SEC profile of in vivo pulldown assay of *Di*Cas7-11 and *Di*TPR-CHAT by size exclusion chromatography using a Superdex200 Increase 10/300 column. **c** SDS-PAGE profile of in vivo pulldown assay of *Di*Cas7-11 and *Di*TPR-CHAT proteins. **d** Workflow of the pulldown assay of *Di*Cas7-11 and *Di*TPR-CHAT in vivo. **e** SEC profile of in *vitro* pulldown assay of *Di*Cas7-11 and *Di*TPR-CHAT by size exclusion chromatography using a Superdex200 Increase 10/300 column. **f** SDS-PAGE profile of the in vitro pulldown assay of *Di*Cas7-11 and *Di*TPR-CHAT proteins. **g** In vitro analysis of cleavage activity using labelled target RNA with crRNA and different assembly order. The system in brackets was treated by incubation at 37 °C for 1 h, the system after brackets was treated by incubation at 37 °C for 30 min. The control consists of the labelled target RNA only. **h** EMSA of *Di*Cas7-11$_{\text{Inactive}}$ with *Di*TPR-CHAT$_{\text{NTD}}$ (NTD), and *Di*TPR-CHAT$_{\text{CTD}}$ (CTD) in the presence of crRNA and labelled target RNA with different assembly order and molar ratio. The system in brackets was incubated first at 37 °C for 1 h, and the system after brackets was further incubated at 37 °C for 30 min.

Figure. S2.

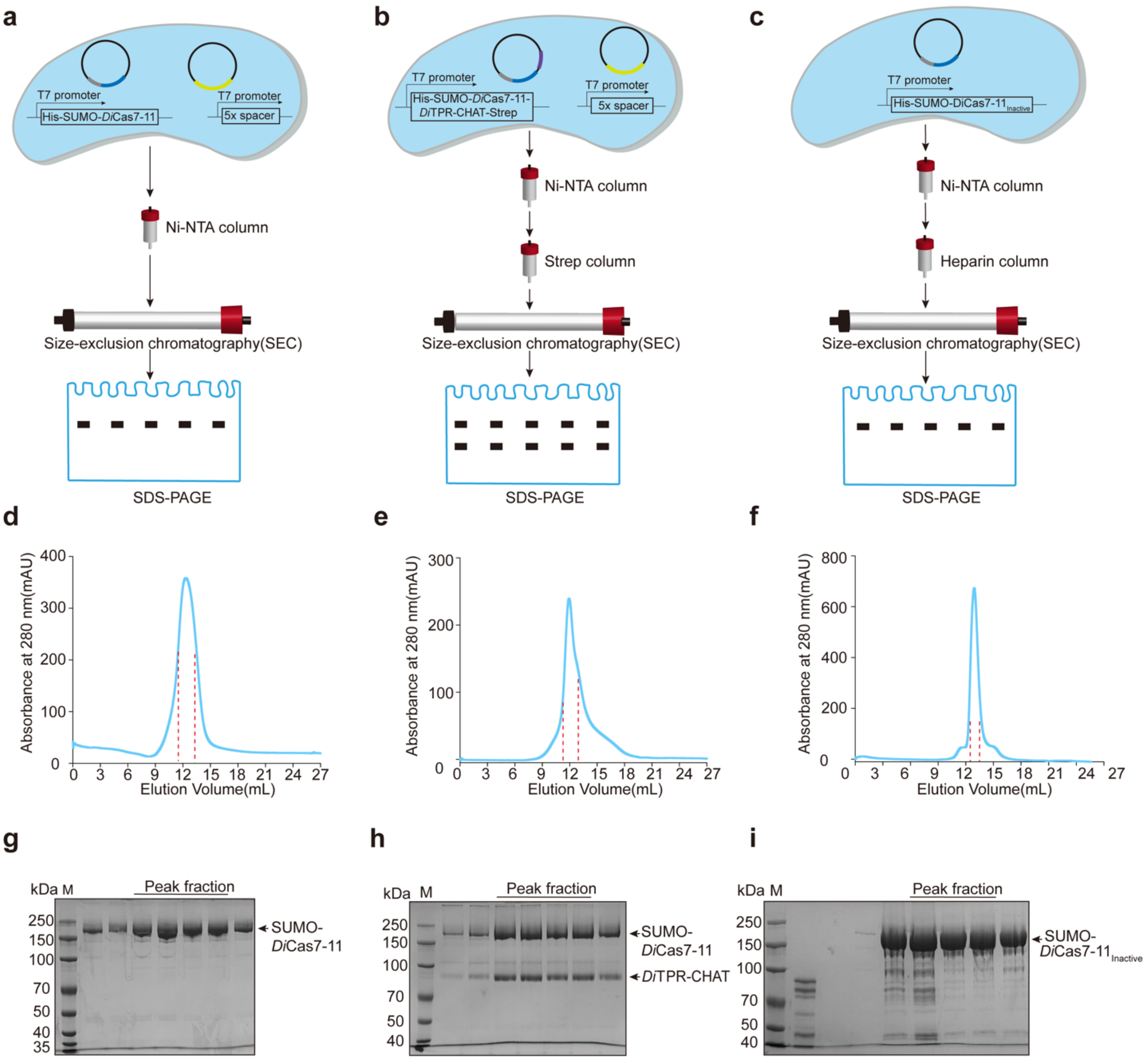

Figure. S2. Expression and purification of *Di*Cas7-11-crRNA, *Di*Cas7-11-crRNA-TPR-CHAT complexes and *Di*Cas7-11$_{\text{Inactive}}$ for cryo-EM. **a-c** Workflow of the expression and purification of *Di*Cas7-11-crRNA (a), *Di*Cas7-11-crRNA-TPR-CHAT (b), and *Di*Cas7-11$_{\text{Inactive}}$ (c), respectively. **d-f** SEC profiles of *Di*Cas7-11-crRNA (d), *Di*Cas7-11-crRNA-TPR-CHAT (e), and *Di*Cas7-11$_{\text{Inactive}}$ (f), respectively. **g-i** SDS-PAGE profiles of *Di*Cas7-11-crRNA (g), *Di*Cas7-11-crRNA-TPR-CHAT (h), and *Di*Cas7-11$_{\text{Inactive}}$ (i), respectively.

Figure. S3.

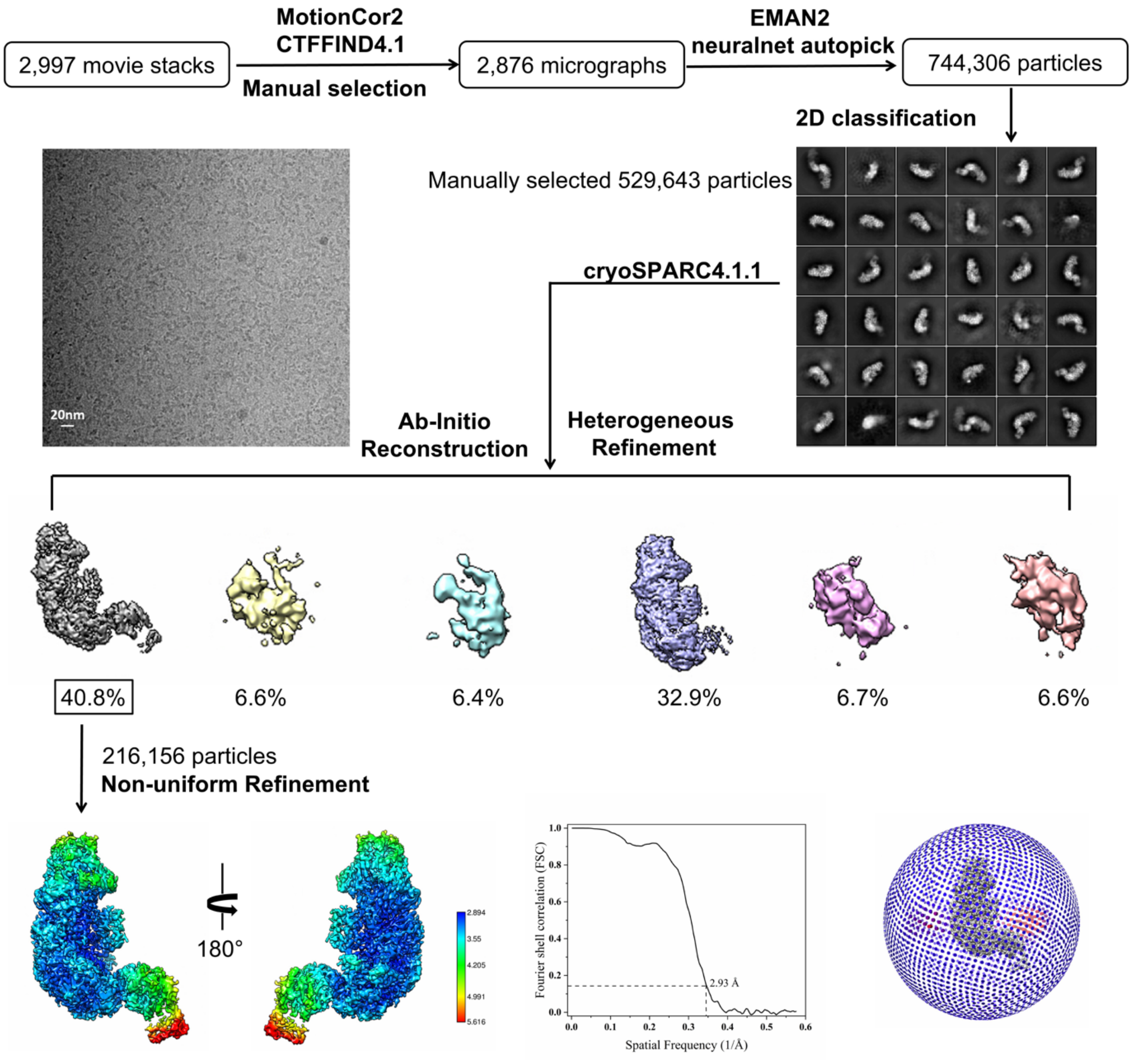

Figure. S3. Cryo-EM image processing and 3D reconstruction for the *Di*Cas7-11-crRNA complex.

Figure. S4.

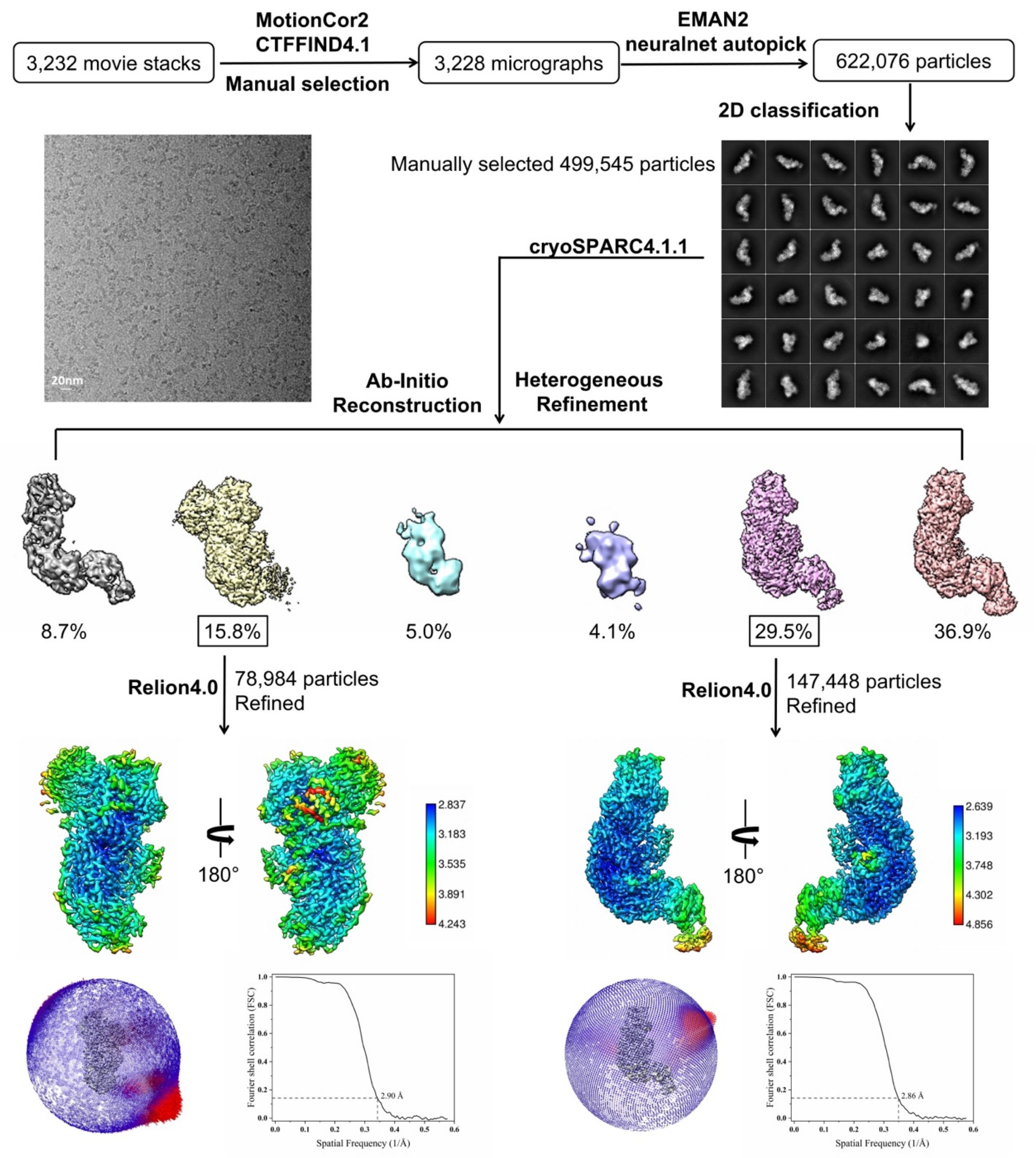

Figure. S4. Workflow of the cryo-EM image processing and 3D reconstruction for the *Di*Cas7-11-crRNA-TPR-CHAT and *Di*Cas7-11-crRNA-TPR-CHAT$_{NTD}$ complexes. **a** Cryo-EM image processing and 3D reconstruction for the *Di*Cas7-11-crRNA-TPR-CHAT and *Di*Cas7-11-crRNA-TPR-CHAT$_{NTD}$ complexes. **b** Cryo-EM map densities of TPR1, L2 (G368-G398), Cas7.4 (V1317-

R1336) in *Di*Cas7-11-crRNA-TPR-CHAT complex, and TPR-CHAT$_{NTD}$ in *Di*Cas7-11-crRNA-TPR-CHAT$_{NTD}$ complex.

Figure. S5.

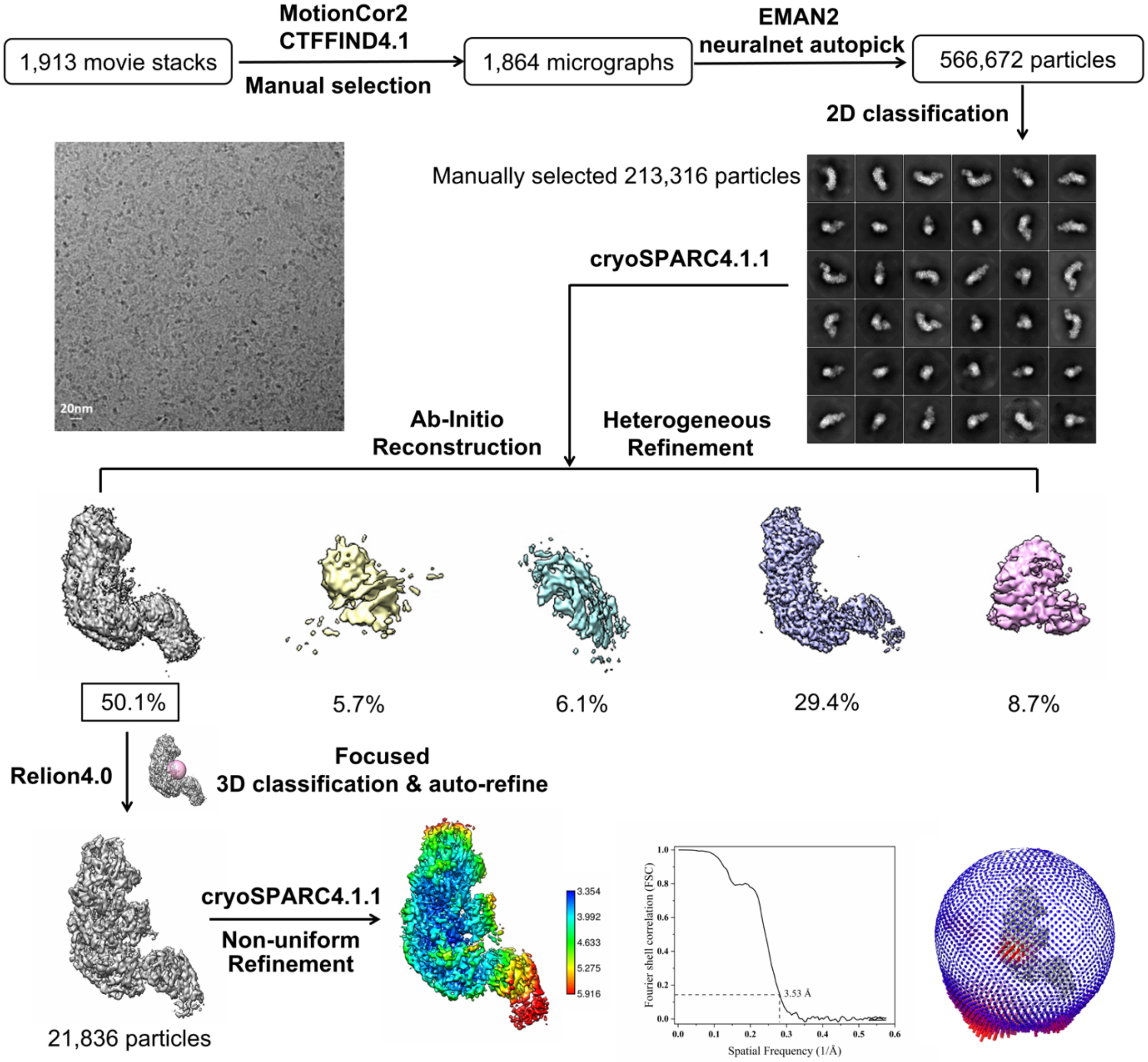

Figure. S5. Cryo-EM image processing and 3D reconstruction for the *Di*Cas7-11$_{\text{Inactive}}$-crRNA complex.

Figure. S6.

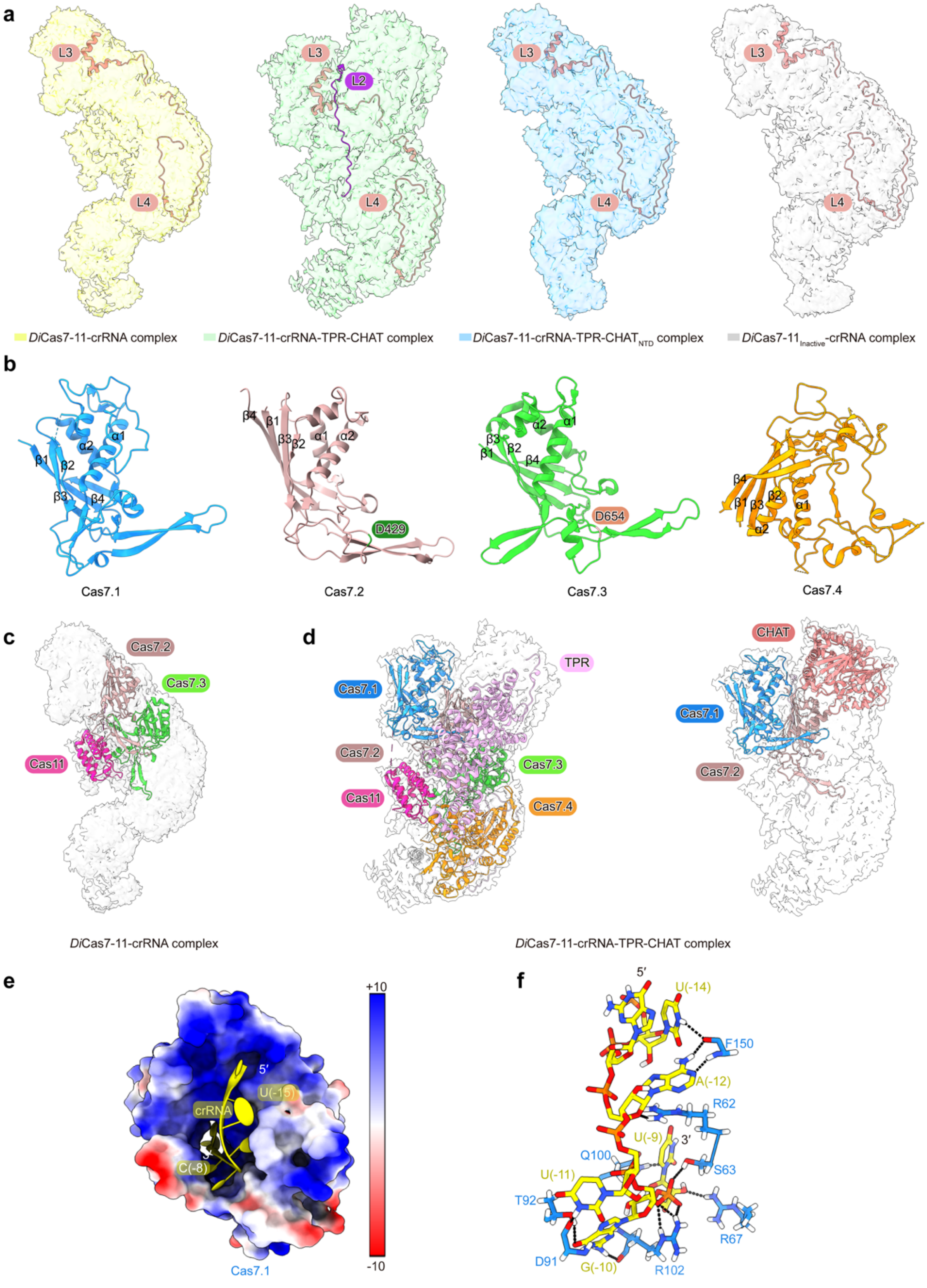
a
L3
L4
L2
DiCas7-11-crRNA complex
DiCas7-11-crRNA-TPR-CHAT complex
DiCas7-11-crRNA-TPR-CHATNTD complex
DiCas7-11Inactive-crRNA complex
b
β1
β2
β3
β4
α1
α2
D429
D654
Cas7.1
Cas7.2
Cas7.3
Cas7.4
c
d
Cas7.2
Cas7.3
Cas11
TPR
Cas7.1
Cas7.4
CHAT
DiCas7-11-crRNA complex
DiCas7-11-crRNA-TPR-CHAT complex
e
+10
-10
5′
U(-15)
crRNA
C(-8)
Cas7.1
f
5′
U(-14)
F150
A(-12)
R62
U(-9)
3′
Q100
S63
U(-11)
T92
R67
D91
G(-10)
R102

Figure. S6. Overall structures of *Di*Cas7-11-crRNA, *Di*Cas7-11-crRNA-TPR-CHAT, *Di*Cas7-11-crRNA-TPR-CHAT$_{NTD}$ and *Di*Cas7-11$_{Inactive}$-crRNA complexes. **a** L3 and L4 in *Di*Cas7-11-crRNA, *Di*Cas7-11-crRNA-TPR-CHAT, *Di*Cas7-11-crRNA-TPR-CHAT$_{NTD}$ and *Di*Cas7-11$_{Inactive}$-crRNA complexes, L2 in *Di*Cas7-11-crRNA-TPR-CHAT complex. **b** Cartoon representations of Cas7.1, Cas7.2, Cas7.3 and Cas7.4 domains. **c** Cartoon representations of Cas7.2, Cas7.3 and Cas11 domains. **d** Cartoon representations of Cas7.1, Cas7.2, Cas7.3, Cas7.4, Cas11, TPR and CHAT domains. **e** Electrostatic surface potential analysis of the interaction region of Cas7.1 with the 5′-repeat region of crRNA (U (-15)–C (-8)). **f** U (-14), A (-12), U (-11), G (-10) and U (-9) in the 5′-repeat region in crRNA form hydrogen bonds with Cas7.1.

Figure. S7.

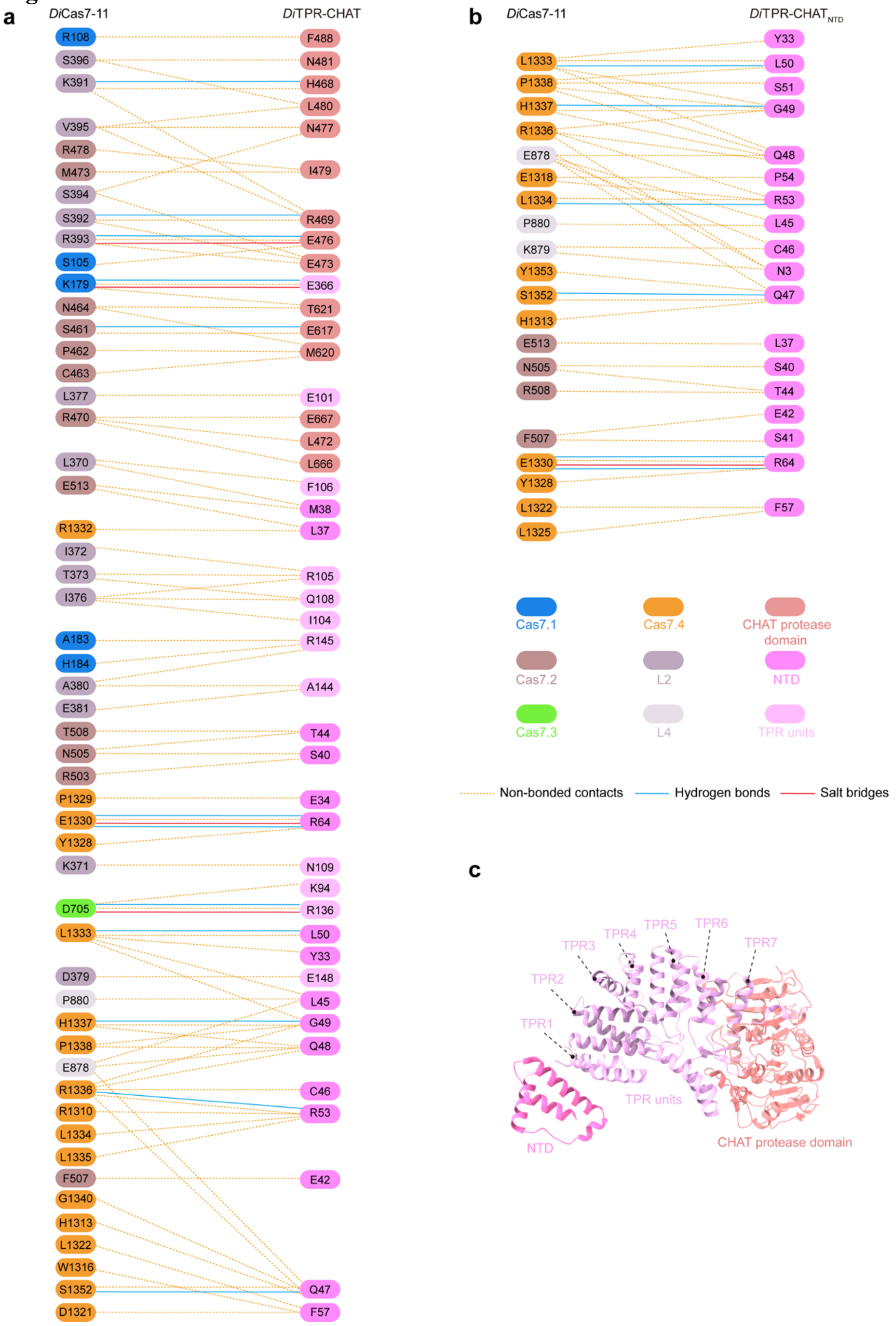
a
DiCas7-11
DiTPR-CHAT
R108
S396
K391
V395
R478
M473
S394
S392
R393
S105
K179
N464
S461
P462
C463
L377
R470
L370
E513
R1332
I372
T373
I376
A183
H184
A380
E381
T508
N505
R503
P1329
E1330
Y1328
K371
D705
L1333
D379
P880
H1337
P1338
E878
R1336
R1310
L1334
L1335
F507
G1340
H1313
L1322
W1316
S1352
D1321
F488
N481
H468
L480
N477
I479
R469
E476
E473
E366
T621
E617
M620
E101
E667
L472
L666
F106
M38
L37
R105
Q108
I104
R145
A144
T44
S40
E34
R64
N109
K94
R136
L50
Y33
E148
L45
G49
Q48
C46
R53
E42
Q47
F57
b
DiCas7-11
DiTPR-CHAT$_{NTD}$
L1333
P1338
H1337
R1336
E878
E1318
L1334
P880
K879
Y1353
S1352
H1313
E513
N505
R508
F507
E1330
Y1328
L1322
L1325
Y33
L50
S51
G49
Q48
P54
R53
L45
C46
N3
Q47
L37
S40
T44
E42
S41
R64
F57
Cas7.1
Cas7.4
CHAT protease domain
Cas7.2
L2
NTD
Cas7.3
L4
TPR units
Non-bonded contacts
Hydrogen bonds
Salt bridges
c
TPR1
TPR2
TPR3
TPR4
TPR5
TPR6
TPR7
TPR units
NTD
CHAT protease domain

Figure. S7. Interaction network of *Di*Cas7-11-crRNA with *Di*TPR-CHAT and *Di*TPR-CHAT$_{NTD}$.
a, b Interaction interface of *Di*Cas7-11-crRNA with *Di*TPR-CHAT (a) and *Di*TPR-CHAT$_{NTD}$ (b).
c Structural features of *Di*TPR-CHAT.

Figure. S8.

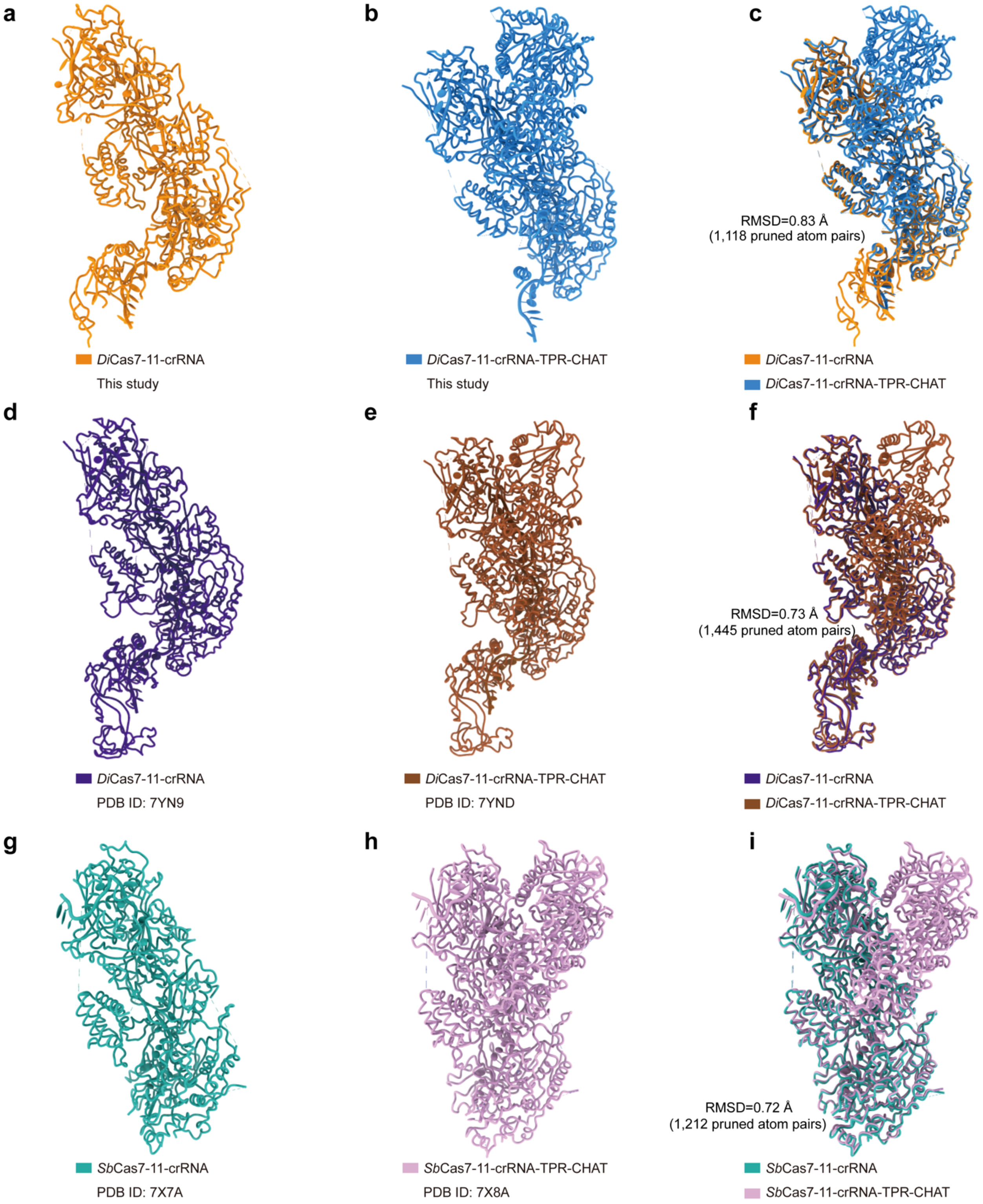
a
DiCas7-11-crRNA
This study
b
DiCas7-11-crRNA-TPR-CHAT
This study
c
RMSD=0.83 Å
(1,118 pruned atom pairs)
DiCas7-11-crRNA
DiCas7-11-crRNA-TPR-CHAT
d
DiCas7-11-crRNA
PDB ID: 7YN9
e
DiCas7-11-crRNA-TPR-CHAT
PDB ID: 7YND
f
RMSD=0.73 Å
(1,445 pruned atom pairs)
DiCas7-11-crRNA
DiCas7-11-crRNA-TPR-CHAT
g
SbCas7-11-crRNA
PDB ID: 7X7A
h
SbCas7-11-crRNA-TPR-CHAT
PDB ID: 7X8A
i
RMSD=0.72 Å
(1,212 pruned atom pairs)
SbCas7-11-crRNA
SbCas7-11-crRNA-TPR-CHAT

Figure. S8. Comparative analysis of Cas7-11-crRNA and Cas7-11-crRNA-TPR-CHAT complexes from different species. **a-c** Cartoon representations of *Di*Cas7-11-crRNA (a), *Di*Cas7-11-crRNA-TPR-CHAT complex (b), and superimposition of *Di*Cas7-11-crRNA and *Di*Cas7-11-crRNA-TPR-CHAT complex (c) in this study. **d-f** Cartoon representations of *Di*Cas7-11-crRNA (PDB ID: 7YN9) (d), *Di*Cas7-11-crRNA-TPR-CHAT complex (PDB ID:7YND) (e), and superimposition of *Di*Cas7-11-crRNA and *Di*Cas7-11-crRNA-TPR-CHAT complex (f). **g-i** Cartoon representations of *Sb*Cas7-11-crRNA (PDB ID: 7X7A) (g), *Sb*Cas7-11-crRNA-TPR-CHAT (PDB ID: 7X8A) (h) and superimposition of *Sb*Cas7-11-crRNA and *Sb*Cas7-11-crRNA-TPR-CHAT complex (i).

Figure. S9.

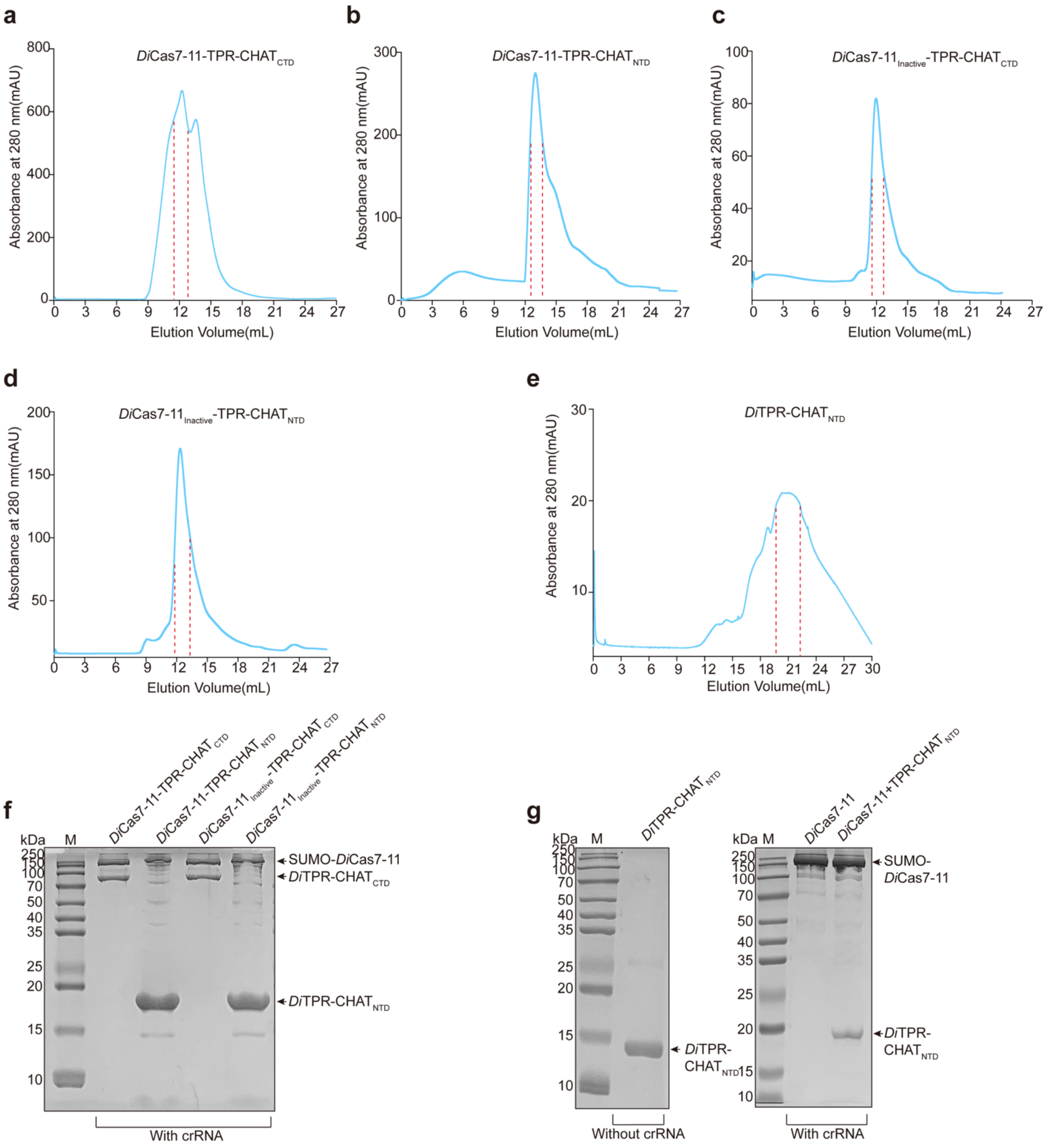

Figure. S9. Protein expression and purification for in vitro nuclease assays and electrophoretic mobility shift assay. **a-e** SEC profiles of *Di*Cas7-11-TPR-CHAT$_{\text{CTD}}$ (a), *Di*Cas7-11-TPR-CHAT$_{\text{NTD}}$ (b), *Di*Cas7-11$_{\text{Inactive}}$-TPR-CHAT$_{\text{CTD}}$ (c), *Di*Cas7-11$_{\text{Inactive}}$-TPR-CHAT$_{\text{NTD}}$ (d), and *Di*TPR-CHAT$_{\text{NTD}}$ (e), respectively. **f-g** SDS-PAGE profile of *Di*Cas7-11-TPR-CHAT$_{\text{CTD}}$,

*Di*Cas7-11-TPR-CHAT$_{NTD}$, *Di*Cas7-11$_{Inactive}$-TPR-CHAT$_{CTD}$, *Di*Cas7-11$_{Inactive}$-TPR-CHAT$_{NTD}$ (f), and *Di*TPR-CHAT$_{NTD}$ (g), respectively.

Figure. S10.

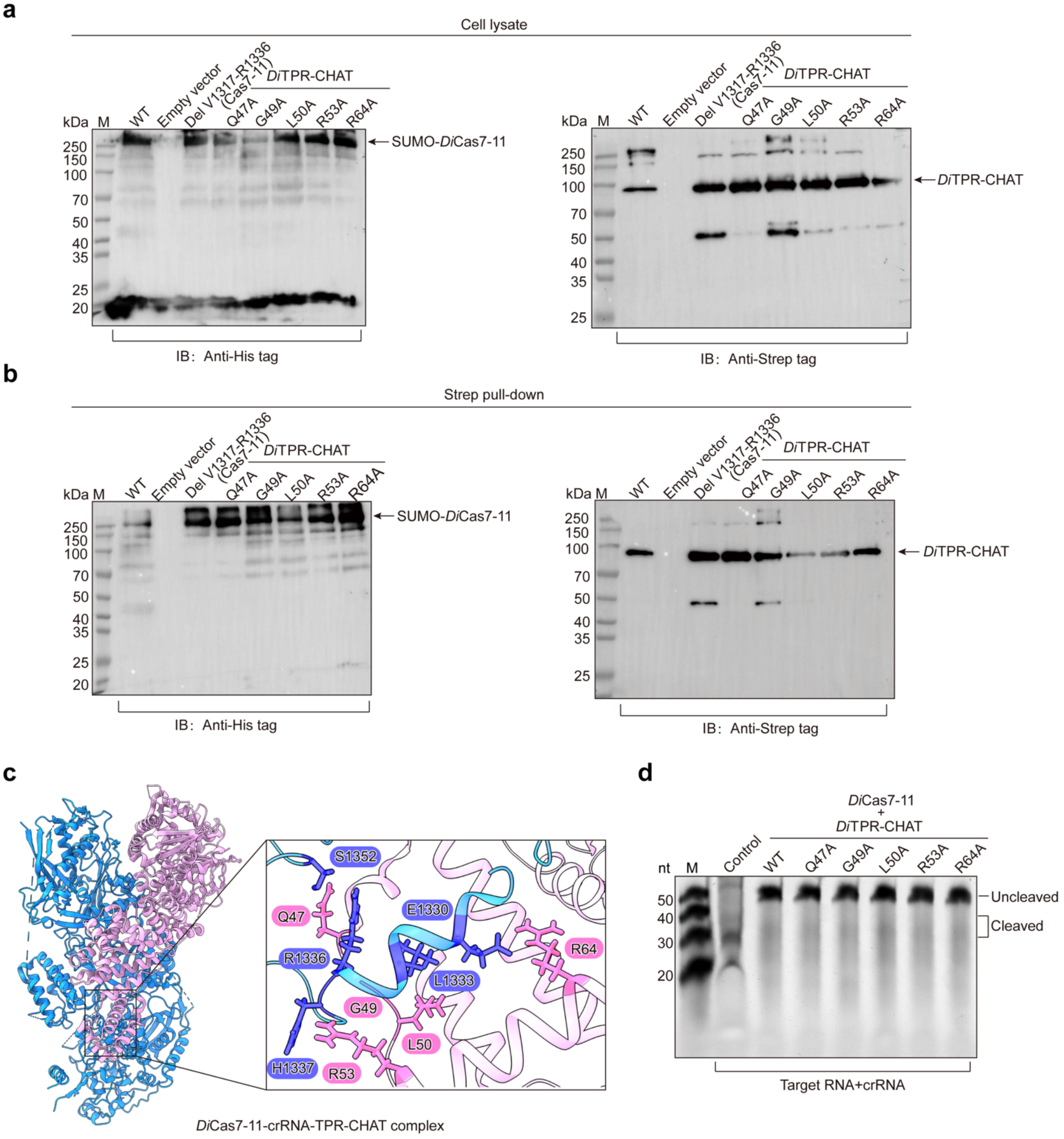

Figure. S10. Interaction analysis between *Di*Cas7-11 and *Di*TPR-CHAT. **a, b** Pull-down assay between *Di*Cas7-11-crRNA and *Di*TPR-CHAT with different mutations (Deletion Cas7.4 (V1371-R1336) and Q47A, G49A, L50A, R53A, R64A in *Di*TPR-CHAT) (n=3). **a** Western-blot analyses of the cell lysates with His tag (Left) and Strep tag (Right). **b** Western-blot analyses of the pulled

samples with His tag (Left) and Strep tag (Right). **c** The hydrogen bonding interaction between Cas7.4 (V1371-R1336) and *Di*TPR-CHAT. **d** In vitro labelled target RNA cleavage analysis with crRNA, *Di*Cas7-11 and *Di*TPR-CHAT (Q47A, G49A, L50A, R53A and R64A). The control consists of *Di*Cas7-11, crRNA and the labelled target RNA.

Figure. S11.

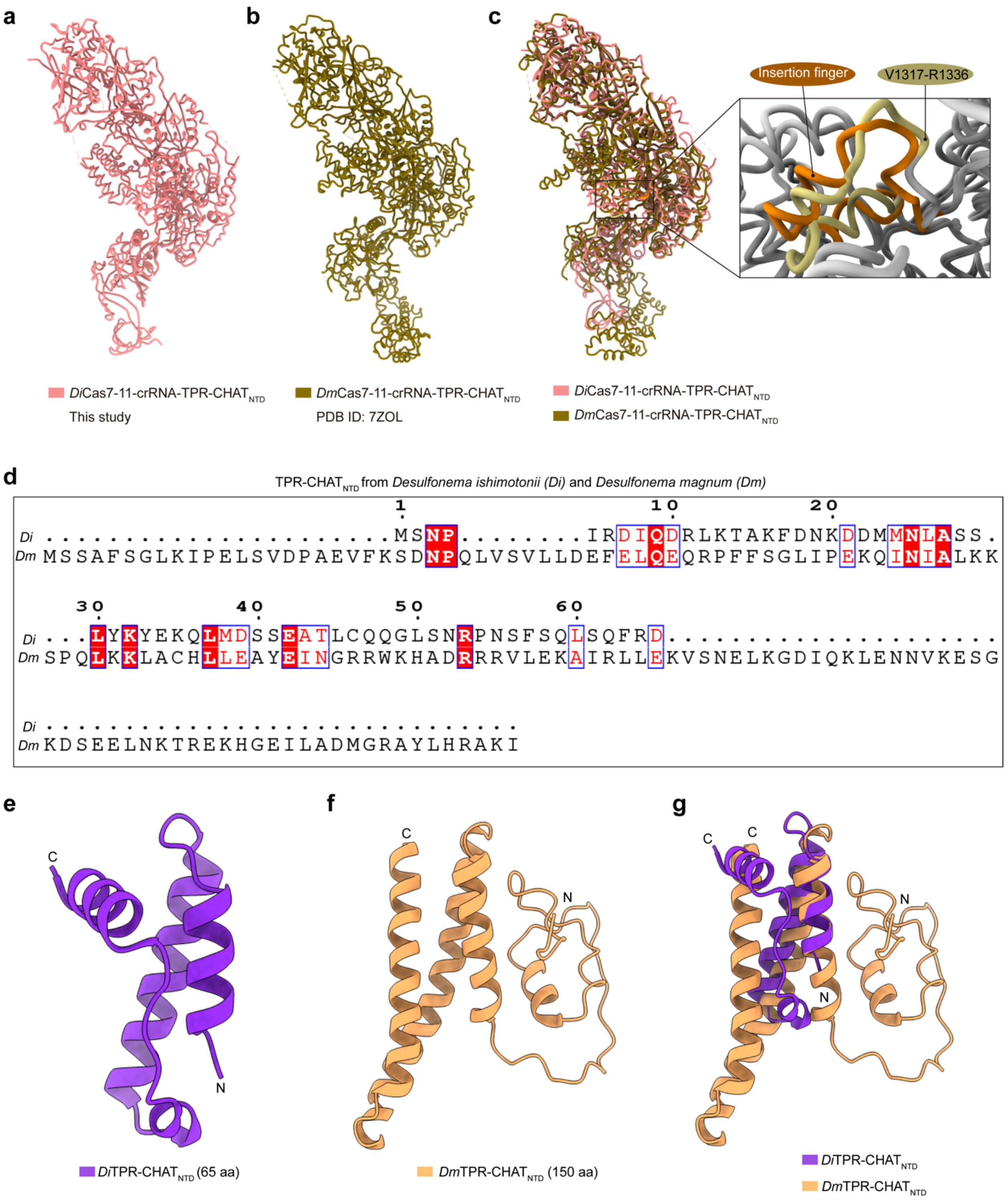

Figure. S11. Structural alignment of TPR-CHAT$_{NTD}$ from different species. **a-c** Cartoon representations of *Di*Cas7-11-crRNA-TPR-CHAT$_{NTD}$ complex (this study) (a), *Dm*Cas7-11-

crRNA-TPR-$CHAT_{NTD}$ complex (PDB ID: 7ZOL) (b), and superimposition of *Di*Cas7-11-crRNA-TPR-$CHAT_{NTD}$ and *Dm*Cas7-11-crRNA-TPR-$CHAT_{NTD}$ complexes (c). High light Cas7.4: V1317-R1336 and insertion finger: C1515-G1543. **d** A sequence alignment of *Di*TPR-$CHAT_{NTD}$ (this study) and *Dm*TPR-$CHAT_{NTD}$ (PDB ID: 7ZOL). **e-g** Cartoon representations of *Di*TPR-$CHAT_{NTD}$ (this study) (e), *Dm*TPR-$CHAT_{NTD}$ (PDB ID: 7ZOL) (f) and superimposition of *Di*TPR-$CHAT_{NTD}$ and *Dm*TPR-$CHAT_{NTD}$ (g).

Figure. S12.

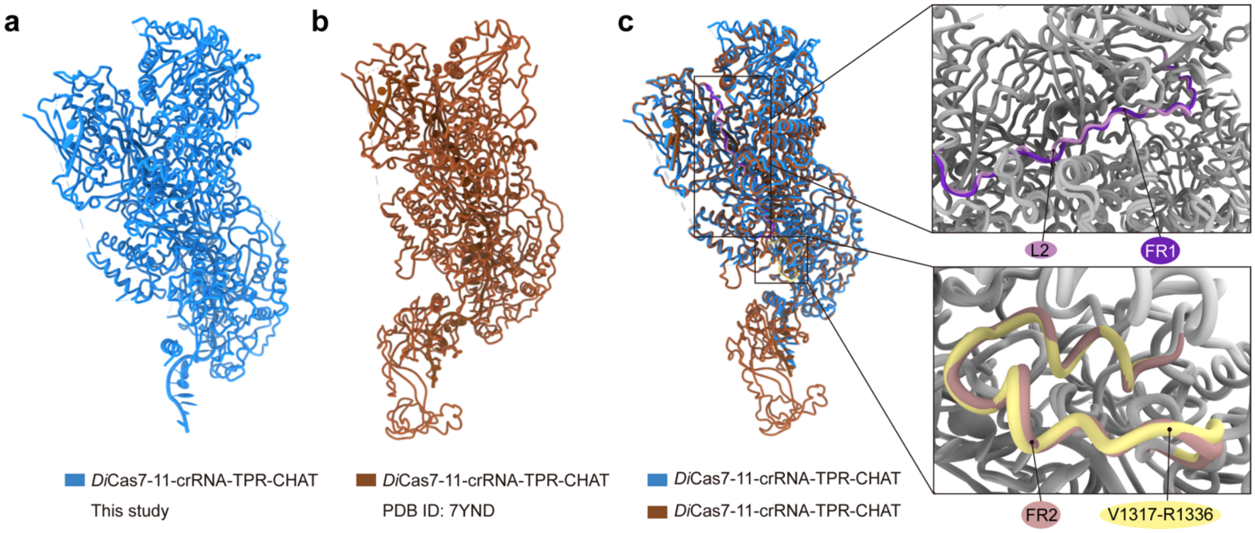

Figure. S12. Structural alignment of Cas7-11-crRNA-TPR-CHAT from *Desulfonema ishimotonii*. **a** Cartoon representation of *Di*Cas7-11-crRNA-TPR-CHAT complex (this study). **b** Cartoon representation of *Di*Cas7-11-crRNA-TPR-CHAT complex (PDB ID: 7YND). c Superimposition of *Di*Cas7-11-crRNA-TPR-CHAT (this study) and *Di*Cas7-11-crRNA-TPR-CHAT (PDB ID: 7YND) complexes. High light L2: G368-G398, Cas7.4: V1317-R1336, FR1: M366-I397 and FR2: W1316-H1337.

Table S1. Cryo-EM data collection, refinement and validation statistics

	*Di*Cas7-11-crRNA (EMDB-37640) (PDB: 8WM4)	*Di*Cas7-11-crRNA-TPR-CHAT (EMDB-37649) (PDB: 8WMC)	*Di*Cas7-11-crRNA-TPR-$CHAT_{NTD}$ (EMDB-37655) (PDB: 8WML)	*Di*Cas7-11(Inactive)-crRNA (EMDB-37653) (PDB: 8WMI)
**Data collection and processing**				
Magnification	165,000	165,000	165,000	165,000
Voltage (kV)	300	300	300	300
Electron exposure ($e^{-}/Å^2$)	61.5	53.7	53.7	56.6
Defocus range (μm)	-1.0 to -1.8	-1.0 to -1.8	-1.0 to -1.8	-1.0 to -1.8
Pixel size (Å)	0.85	0.85	0.85	0.85
Symmetry imposed	C1	C1	C1	C1
Initial particle images (no.)	529,643	499,545	499,545	213,316
Final particle images (no.)	216,156	78,984	147,448	21,836
Map resolution (Å)	2.93	2.90	2.86	3.53
FSC threshold	0.143	0.143	0.143	0.143
Map resolution range (Å)	2.8-5.6	2.8-4.2	2.6-4.8	3.3-5.9
**Refinement**				
Initial model used	7Y9X	7Y9X	7Y9X	7WAH
Model resolution (Å)	3.3	3.2	3.1	3.9
FSC threshold	0.5	0.5	0.5	0.5
Model resolution range (Å)	2.8-3.4	2.8-3.2	2.8-3.2	3.4-4.0
Map sharpening *B* factor ($Å^2$)	-85.5	-75.3	-82.4	-66.6
Model composition				
Non-hydrogen atoms	22550	33169	25862	21008
Protein residues	1334	1983	1543	1237
Nucleotides	38	38	38	39
Ligands	4	4	4	4
B factors ($Å^2$, mean)				
Protein	77.12	76.44	55.53	101.93
Nucleotide	77.90	81.41	53.47	106.02
Ligand	147.35	93.94	101.06	151.90
R.m.s. dSiations				
Bond lengths (Å)	0.004	0.003	0.003	0.003
Bond angles (°)	0.693	0.606	0.623	0.648
Validation				
MolProbity score	1.53	1.03	1.20	1.49
Clashscore	3.28	1.78	2.15	3.33
Poor rotamers (%)	0	0	0	0
Ramachandran plot				
Favored (%)	93.87	97.54	96.72	94.73
Allowed (%)	6.13	2.46	3.28	5.27
Disallowed (%)	0	0	0	0

The original uncropped Western blots

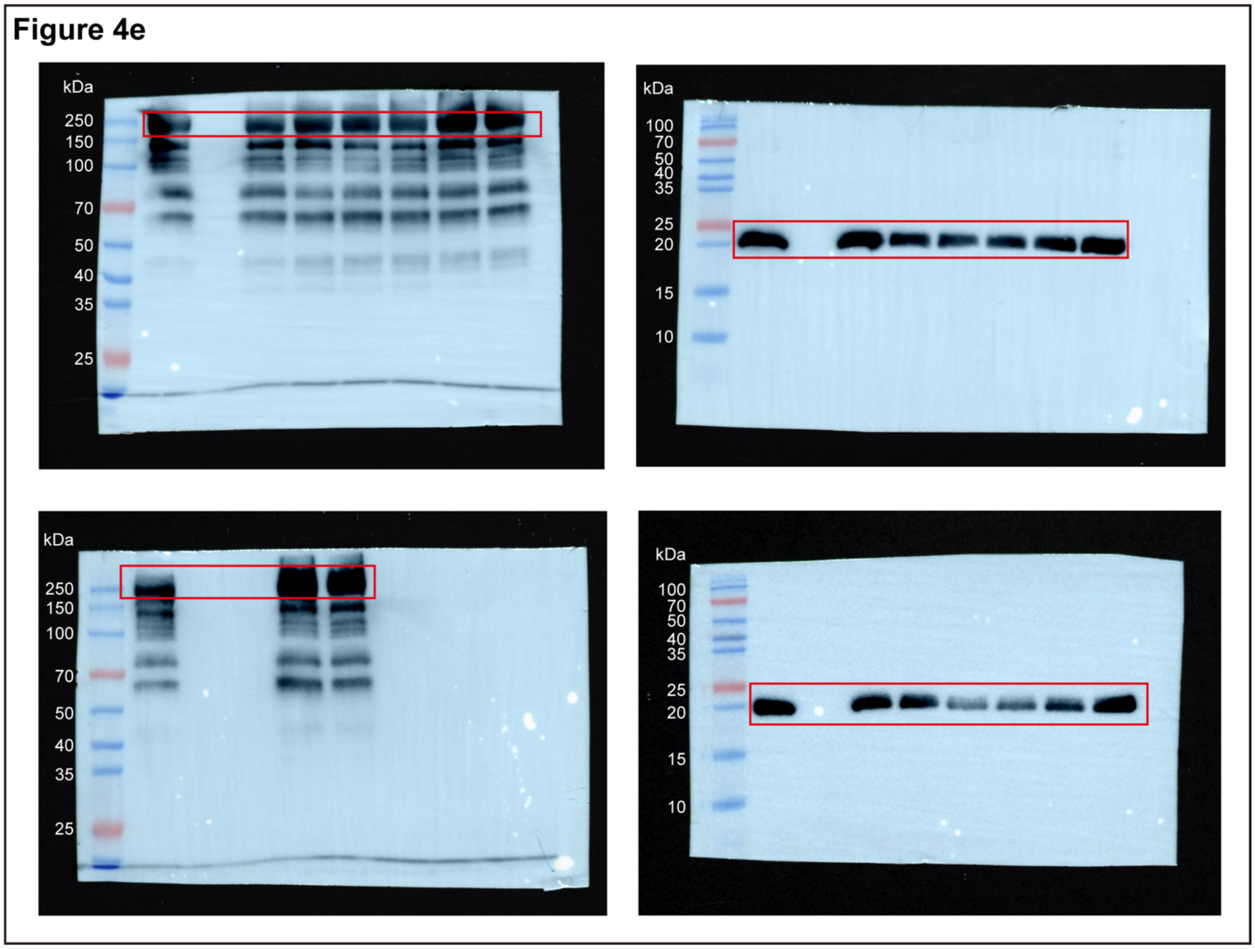

Figure S10

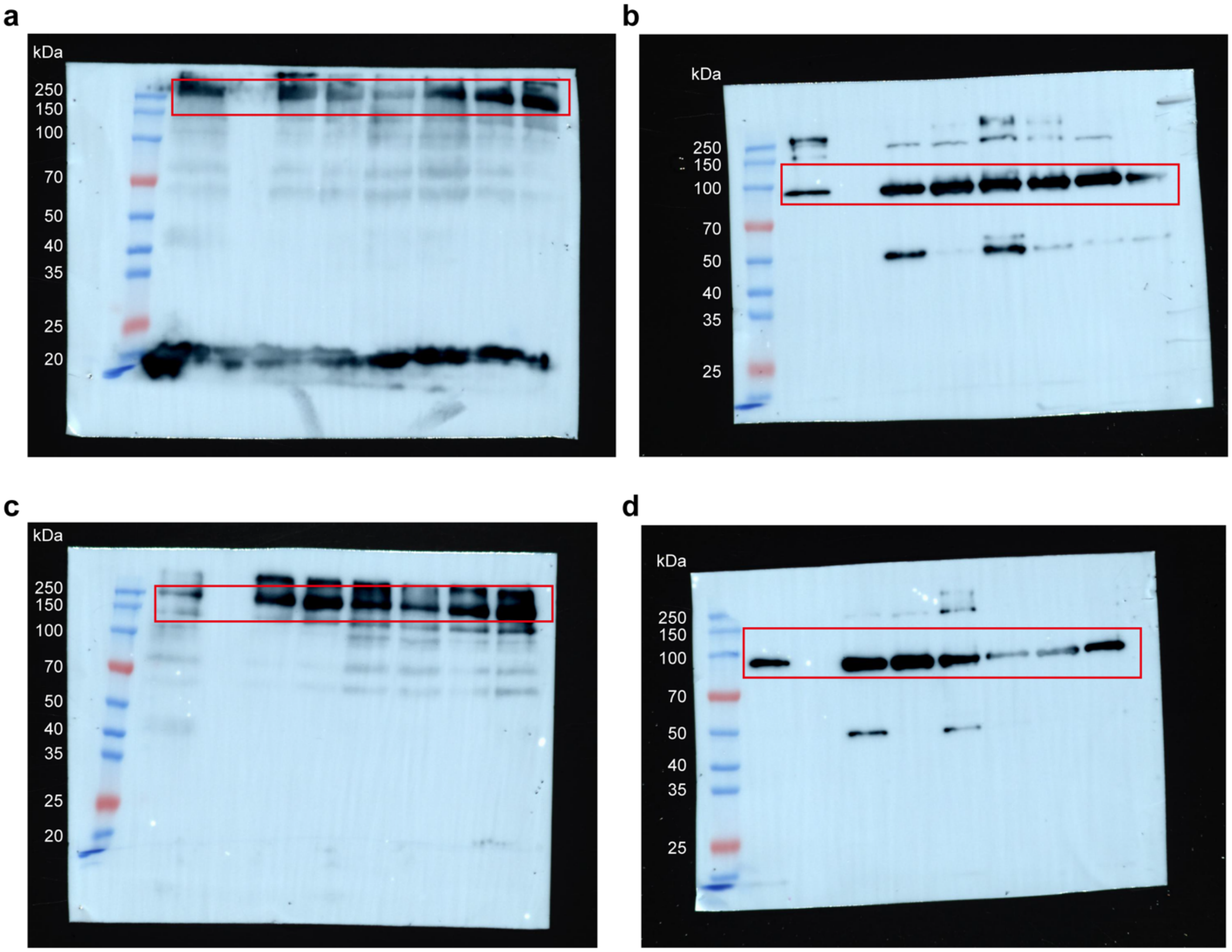
a
kDa
250
150
100
70
50
40
35
25
20
b
kDa
250
150
100
70
50
40
35
25
c
kDa
250
150
100
70
50
40
35
25
20
d
kDa
250
150
100
70
50
40
35
25
